# Distinct Effects of p19 RNA Silencing Suppressor on Small RNA Mediated Pathways in Plants

**DOI:** 10.1371/journal.ppat.1005935

**Published:** 2016-10-06

**Authors:** Levente Kontra, Tibor Csorba, Mario Tavazza, Alessandra Lucioli, Raffaela Tavazza, Simon Moxon, Viktória Tisza, Anna Medzihradszky, Massimo Turina, József Burgyán

**Affiliations:** 1 National Agricultural Research and Innovation Centre, Agricultural Biotechnology Institute, Gödöllő, Hungary; 2 Szent István University, Gödöllő, Hungary; 3 Italian National Agency for New Technologies, Energy and Sustainable Economic Development (ENEA), C.R. Casaccia, Rome, Italy; 4 School of Biological Sciences, University of East Anglia, Norwich, Norfolk, United Kingdom; 5 National Research Council, Institute for Sustainable Plant Protection, Torino, Italy; Institute of Microbiology, CHINA

## Abstract

RNA silencing is one of the main defense mechanisms employed by plants to fight viruses. In change, viruses have evolved silencing suppressor proteins to neutralize antiviral silencing. Since the endogenous and antiviral functions of RNA silencing pathway rely on common components, it was suggested that viral suppressors interfere with endogenous silencing pathway contributing to viral symptom development. In this work, we aimed to understand the effects of the tombusviral p19 suppressor on endogenous and antiviral silencing during genuine virus infection. We showed that ectopically expressed p19 sequesters endogenous small RNAs (sRNAs) in the absence, but not in the presence of virus infection. Our presented data question the generalized model in which the sequestration of endogenous sRNAs by the viral suppressor contributes to the viral symptom development. We further showed that p19 preferentially binds the perfectly paired ds-viral small interfering RNAs (vsiRNAs) but does not select based on their sequence or the type of the 5’ nucleotide. Finally, co-immunoprecipitation of sRNAs with AGO1 or AGO2 from virus-infected plants revealed that p19 specifically impairs vsiRNA loading into AGO1 but not AGO2. Our findings, coupled with the fact that p19-expressing wild type *Cymbidium ringspot virus* (CymRSV) overcomes the *Nicotiana benthamiana* silencing based defense killing the host, suggest that AGO1 is the main effector of antiviral silencing in this host-virus combination.

## Author Summary

To better understand the specific effect of p19 viral suppressor of RNA silencing (VSR) on antiviral silencing and endogenous small RNA pathways, we generated a *N*. *benthamiana* plant (p19syn) capable of sustaining the ectopic expression of the *Cymbidium ringspot virus* (CymRSV) p19 upon infection with a suppressor-deficient CymRSV (Cym19stop). By using wt and p19syn plants in combination with CymRSV and Cym19stop, we were able to analyze the effects of p19 provided “in trans” and “in cis” during the viral invasion of the plant. We have shown that p19 can efficiently sequester endogenous small RNAs (sRNAs) in mock-inoculated p19syn plants while it does not bind these sRNAs upon Cym19stop infection. Also, the presence of p19 in virus infection did not alter the expression of miRNAs significantly. These findings do not support the widely accepted assumption that viral symptoms are the direct consequence of the impact of VSRs on endogenous silencing pathways. We demonstrated that p19 preferentially sequesters positive:negative viral short interfering RNAs (vsiRNAs) pairs and that the binding by p19 is independent of vsiRNA sequence or the type of the 5’-end nucleotide. We have also found that 3’ truncation is induced on p19 bound sRNAs. Finally using AGO1- and AGO2- immunoprecipitation experiments we observed that p19 specifically compromises vsiRNAs’ loading into AGO1 but not AGO2. Since antiviral silencing is strongly inhibited by p19, this suggests that AGO1 is the main effector protein against CymRSV tombusvirus.

## Introduction

Viruses are among the most important plant pathogens that cause huge economic losses in many agriculturally important crops worldwide. The invasion of the host by viruses deeply alters the physiology of the plants at cellular and tissue levels due to the interaction of the virus with the cellular pathways, which ultimately leads to viral symptom development. During evolution, plants have developed diverse strategies to combat virus infections. Amongst others, RNA silencing is one of the most important mechanisms that serve to fight against viruses [1,2].

RNA silencing is a conserved eukaryotic pathway involved in almost all cellular processes like development, stress responses and antiviral defense. RNA silencing relays on the 21–24 nt short interfering RNAs (siRNAs) or micro RNAs (miRNAs) the hallmark molecules of silencing [3]. The siRNAs and the miRNAs (collectively named small RNAs, sRNAs) are processed by RNase III-type ribonucleases, the DICER (in plants Dicer-Like, DCL) enzymes [4,5] in collaboration with their partner DOUBLE-STRANDED RNA BINDING (DRB) proteins [6–9]. sRNAs are 2’-O-methylated by HUA ENHANCER1 (HEN1) at their 3’ protruding ends [10], a reaction that serves to protect them against poly-uridylation and subsequent degradation [11]. sRNAs then associate with ARGONAUTE (AGO) proteins [12–14] the central effectors of RNA-induced silencing complex (RISC) [15,16]. Based on the sequence complementarity, sRNAs guide RISC to silence cognate RNAs through cleavage or translational repression (post-transcriptional gene silencing, PTGS) or induce chromatin/DNA modifications of the specific genomic locus (transcriptional gene silencing, TGS) [17–19]. In some specific cases, amplification of silencing occurs through double-stranded RNA synthesis by RNA-dependent RNA polymerases (RDRs) and secondary siRNA production [20–22]. sRNAs are non-cell autonomous, they can move within the plant to transmit gene silencing from cell-to-cell or systemically on long distance as mobile silencing signals [23–25].

Players of the antiviral silencing overlap with those of the endogenous silencing pathway [1,26]. Antiviral RNA silencing is triggered by the presence of viral dsRNA structures such as replication intermediates or intra—molecular fold—back structures of the invading virus. These dsRNA structures are processed by DCL4 or DCL2, to produce viral short interfering RNAs (vsiRNAs) [2,3,27–32]. The vsiRNAs guide self-silencing of the parental viral genomic RNA as part of the antiviral response through the action of AGO effectors [1,13]. Among the AGOs implicated in antiviral defense, AGO1 and AGO2 were identified as the most important players. AGO1 was shown to have antiviral roles against a number of viruses in *A*. *thaliana* [33–36], *N*. *benthamiana* [26,37,38] and in rice [39]. AGO2 was found to be important in the antiviral silencing response in *A*. *thaliana* [35,36,40–45]. In *N*. *benthamiana*, the important model organism for plant virology studies, AGO2 was proposed to protect against *Potato virus X* [46] and the suppressor-deficient *Tomato bushy stunt virus* (TBSV) [47,48]. However, recent observation suggested that AGO1 constitutes a solid layer of defense against tombusvirus infections [49]. As AGO1 is the negative regulator of AGO2, it is believed that AGO2 represents a second layer of antiviral defense [40].

Viruses, to counteract host defense, have evolved viral suppressors of RNA silencing (VSRs) providing strong evidence for the antiviral nature of silencing [1,50,51]. Most viruses studied so far were found to encode at least one VSR. VSRs were shown to block silencing at multiple steps like initiation, effector complex assembly, silencing amplification but also through transcriptional control of endogenous factors, hormone signal modulation or interaction with protein-based immunity [51,52]. The absence or inactivation of VSRs leads to the recovery of plants from viral infections, demonstrating the effect of plant antiviral silencing response [53–55]. Although several VSRs have been identified in the past, our knowledge about the precise molecular basis of their action and their multifunctional roles have only been resolved in a few cases [1].

The p19 protein of tombusviruses is one of the best-known VSR. Crystallographic studies have shown that p19 tail-to-tail homodimer acts as a molecular caliper to size-select and sequester siRNA duplexes in a sequence-independent manner [56–58] preventing the loading of siRNAs into AGO effector proteins [59,60]. Based on p19 expressing transgenic plants it was proposed that during virus infection p19 efficiently prevents miRNAs loading into RISC deeply compromising the endogenous miRNA pathways of the plants [61–63]. In particular, it was reported that three distinct VSRs (HcPro, p19 and P15) compromised the regulation of the miR167 target AUXIN RESPONSE FACTOR 8 (ARF8) when constitutively expressed in transgenic plants [64]. It was also proposed that misregulation of miR167 is the major cause for the developmental aberrations exhibited by VSR transgenic plants and for the phenotypes induced during viral infections [64]. Contradictory to these, other data suggests that during genuine *Cymbidium ringspot virus* (CymRSV) infections miRNA sequestration by p19 is very poor and may depend on spatial and temporal co-expression of miRNA duplex and the VSR [65]. vsiRNAs but not miR159 were shown to be sequestered efficiently into p19-homodimer:siRNA nucleoprotein complex, whereas miR159 was efficiently incorporated into RISC complex [66,67]. These findings suggest that, in virus-infected plants, p19 potently affects vsiRNA-pathway and at the much lesser extent the miRNA one. Besides, independently of its siRNA binding capacity, p19 similarly to other VSRs can promote miR168 transcriptional induction that results in miR168-guided AGO1 down-regulation [66,68]. Thus, the interaction of p19 with endogenous silencing pathways and its contribution to viral symptom development is far from being fully uncovered.

To better understand the impact of p19 on silencing and its role in viral symptom development we employed a synthetic p19-expressing transgenic *N*. *benthamiana* plant line (p19syn) or wild type plant as control in combination with its wild-type (CymRSV) or suppressor deficient virus (Cym19stop) infection: (i) wt virus infection of wt *N*. *benthamiana* (p19 in “cis”), (ii) Cym19stop-infection in wt *N*. *benthamiana* (virus present, no suppressor), (iii) Cym19stop-infection in p19syn plants (p19 in “trans”) and (iv) p19syn plants (p19 only). In this way we were able to compare the impact of p19 on its own or in the genuine virus-infected background.

We analyzed p19 ability to sequester vsiRNAs and plant endogenous sRNAs, with and without viral background. Consistently with our previous results, we have found that p19 can bind vsiRNAs when expressed either “in cis” (from the CymRSV wild-type virus, in wild-type *N*. *benthamiana*) or “in trans” (transgenically expressed in p19syn plants infected with the suppressor-deficient Cym19stop virus). In line with our previous findings, p19 efficiently bound endogenous sRNA duplexes only in the absence of the virus infection, suggesting that p19 impact on endogenous pathways is restricted. Analyzing the siRNA pool immunoprecipitated by p19 through high-throughput sequencing, we found that p19 changes the bias of positive vsiRNAs towards a more equilibrated positive/negative strand ratio, suggesting a preference for perfect ds-vsiRNAs. We also showed that p19 prevents mainly AGO1 but not AGO2 loading with vsiRNAs. This finding suggests a key role of AGO1 opposed to AGO2 during the antiviral response.

## Results

### Generation of synthetic p19 expressing (p19syn) transgenic *N*. *benthamiana* plants

**Fig 1:**
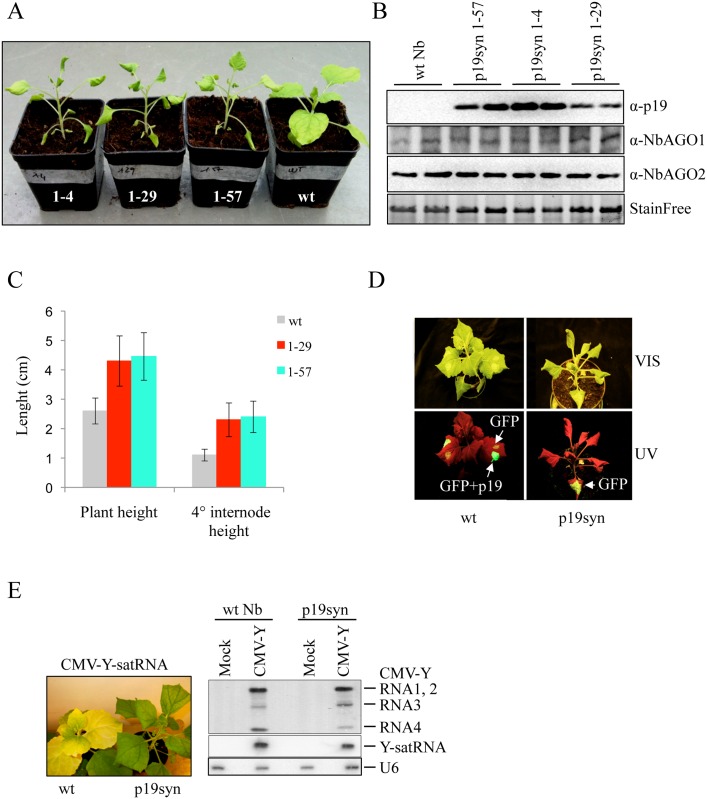
p19 protein expressing transgenic *Nicotiana benthamiana* (p19syn) plants. (A) Specific developmental phenotype of p19syn plants compared to wild type plants. Three representative independent transgenic lines are shown alongside wild-type *N*. *benthamiana* plant. (B) Western blot of p19syn transgenic and wild-type plants from lines shown in (A). The presence of p19 transgene does not impact NbAGO1 or NbAGO2 protein levels. Protein loading is shown below (StainFree). (C) Stem length from cotyledons to the fourth and last leaf insertion in wt and T1 p19syn plants at 8–9 leaf stage (lines:1–29 and 1–57) were used to show the elongated internode trait of p19syn plants. Bars: standard deviation. (D) Wild type and p19syn plants agroinfiltrated with GFP and GFP+p19 constructs as indicated. Pictures were taken at 4 dpi. (E) p19-mediated inhibition of RNA silencing of tobacco magnesium protoporphyrin chelatase subunit I *(ChlI)* (a key chlorophyll biosynthetic gene) induced by CMV + Y-satRNA infection (14 dpi, left panel), silencing of *ChlI* causes yellowing on wt plants. Northern blot hybridization of CMV + Y-satRNA infected wt or p19syn plants; nucleolar small RNA U6 was used as an internal control (right panel).

To uncouple p19 effects elicited by virus infection on RNA silencing and host plant symptom development we prepared p19-expressing *N*. *benthamiana* plants (Fig 1A–1C). To avoid the interference between the *p19* transgene and the challenging virus (p19-deficient, Cym19stop), we modified the *p19* transgene introducing all possible silent nucleotide changes. In this way, we reduced the nucleotide sequence similarity between the transgene and the challenging virus to 68% while keeping the amino acid identity at 100% (S1 Fig). These plants were named synthetic-p19 expressing plants (p19syn). The p19syn plants showed strong phenotype characterized with elongated stem internodes and typical leaf distortions (Fig 1A, 1C and 1D and S2A Fig) suggesting that the expressed p19 protein retained it suppressor activity, thus potentially compromising the endogenous silencing pathways. Importantly, this phenotype was clearly different from that of virus-infected stunted dwarf plant (S2A Fig). Transgenic line 1–57 was selected for further studies (Fig 1A and 1B). First we tested the silencing suppressor activity of transgenically expressed p19 in a GFP transient assay (see Materials and Methods). When GFP sense transgene was transiently expressed in wild-type plant leaves, spontaneously triggered silencing almost completely diminished GFP expression at four days post infiltration. In contrary when GFP was expressed in p19syn plants its expression was still strong as visualized under UV light (Fig 1D). The lack of GFP silencing in p19syn plants confirmed the suppressor activity of the p19 transgene. Next we tested p19 suppressor activity in an authentic virus infection context: we challenged the p19syn plants by the infection with *Cucumber mosaic virus + yellow satellite RNA* (CMV + Y-satRNA). CMV + Y-satRNA was reported to induce bright yellow symptoms on *N*. *benthamiana* through targeting the tobacco magnesium protoporphyrin chelatase subunit I (*ChlI*) gene involved in chlorophyll biosynthesis by Y-satRNA derived siRNA [69]. The CMV-Y-satRNA infected wt *N*. *benthamiana* plants developed the bright yellow symptoms while the infected p19syn plants failed to show the typical yellowing (Fig 1E). All these confirmed that the transgenically expressed p19 works as a silencing suppressor *in vivo*.

### p19 preferentially sequesters vsiRNAs but not endogenous sRNAs during virus infection

It is generally assumed that virus encoded suppressors strongly interfere with the endogenous silencing pathway and are central players in the development of viral symptoms [1,61–64]. However, this notion mostly comes from studies that used VSR-expressing transgenic plants without analyzing the effect of the VSRs in an authentic virus infection background. To reinvestigate this dogma we set up an experiment in which we could compare p19 effects (vsiRNA and endogenous sRNA binding) with or without its parental virus infection background. We compared the sRNA binding capability of p19 both in mock- and Cym19stop-inoculated p19syn plants. This setup allowed us to analyze the impact of p19 when provided “in trans” during virus infection. It is worth noting that the suppressor mutant Cym19stop virus in infected p19syn plant was able to invade whole leaves similarly to the CymRSV in wt plants (S2B Fig). In contrast, in the absence of p19, the Cym19stop virus is restricted to the veins [70] (S2B Fig). Besides this, we also inoculated wt plants with CymRSV to study p19 activity “in cis“. Based on previous studies [61,62] we expected p19 to bind ds-sRNAs of both plant and viral origin. p19 immunoprecipitations (IP) were prepared from pooled systemically-infected leaves of virus-infected plants and the corresponding mock-inoculated leaves of p19syn plants. cDNA libraries of sRNAs were generated using RNA samples isolated from inputs and p19 IPs. After quality control filtering and processing steps (see Materials and Methods), sequences flanked by the 3’ and 5’ Solexa adaptors, and ranging in length from 16 to 28 nt, were aligned to the *N*. *benthamiana* and the CymRSV genomes, respectively [71,72].

**Fig 2:**
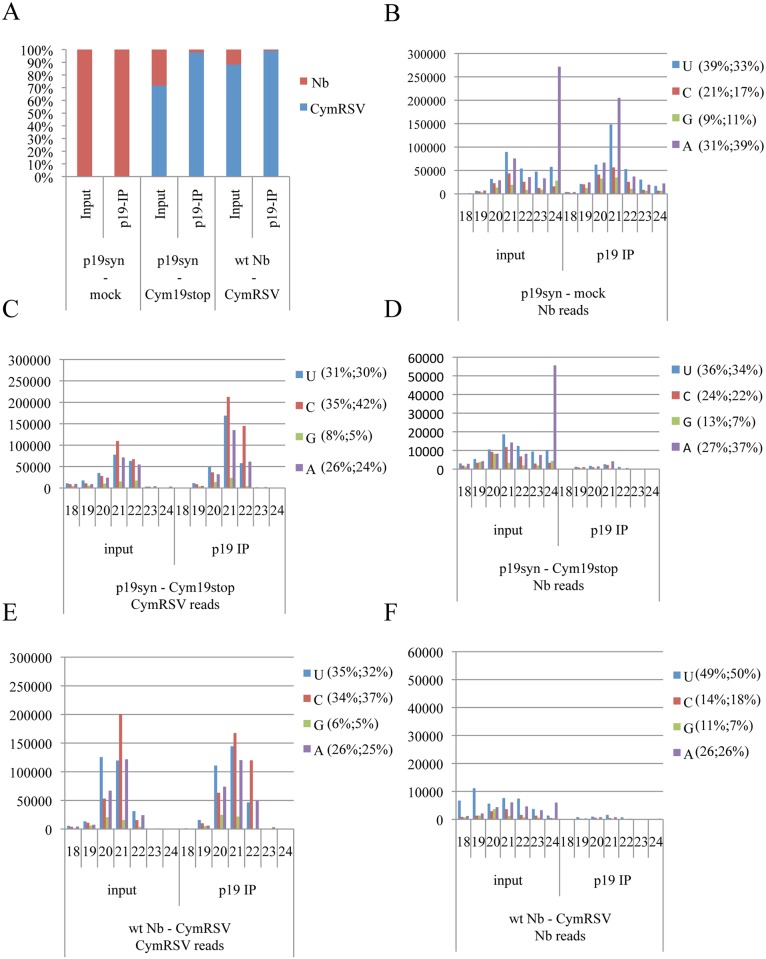
Endogenous and viral sRNAs sequestration preferences of p19. (A) Total and p19-bound vsiRNAs and endogenous sRNAs derived from *N*. *benthamiana* and p19syn plants infected with wt (CymRSV) and suppressor deficient Cym19stop viruses and mock-inoculated plants. (B) input and p19-bound endogenous sRNA duplexes in mock-inoculated plants. (C-F) inputs and p19-bound vsiRNAs and endogenous sRNAs when p19 was expressed in *trans* (C and D) or *in cis* (E and F) during virus infections as indicated. The size classes of sRNAs between 18 and 24nt are indicated by numbers The 5’ nucleotides are indicated by color codes. The percentages of specific 5’-nucleotide sRNAs in input and p19 IP are shown in brackets at the right side of the panel (input%; IP%). Note that B, C, E scales differ from D and F ones. Read counts were normalized to 1 million reads.

**Fig 3:**
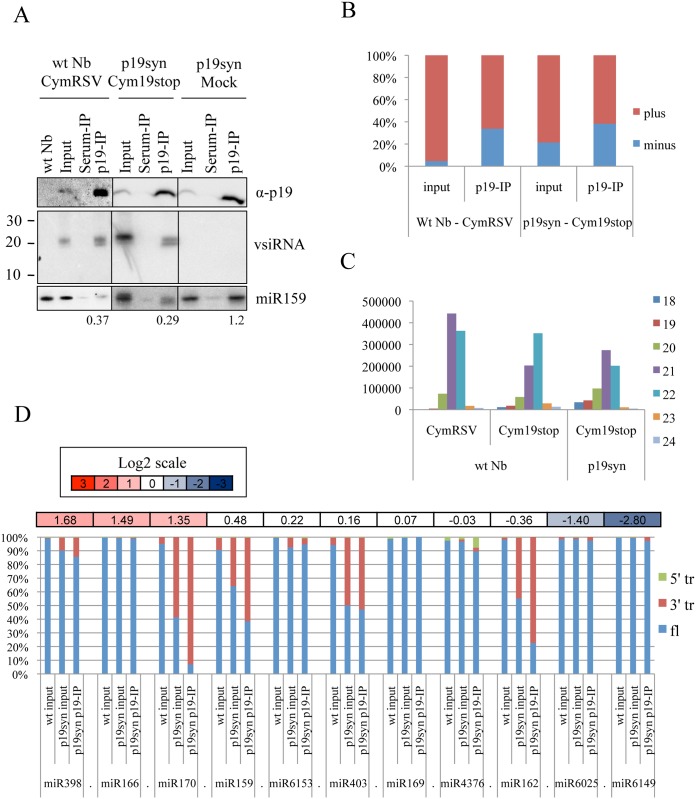
p19 interaction with sRNAs *in vivo*. (A) Western blots of p19 (upper panel), Northern blot of p19-bound vsiRNAs (middle panel) and miR159 (lower panel) during the CymRSV and Cym19stop virus infection of p19syn and wt *N*. *benthamiana* plants as indicated. The ratio of p19-bound miR159 to the input (IP/input) is indicated below the panels. (B) The percent of vsiRNA sequences derived from the positive and negative viral strands in the input and in p19-IP. (C) Global accumulation of different size classes of vsiRNAs in p19syn and wt *N*. *benthamiana* plants infected by CymRSV and Cym19stop as indicated. Size classes of vsiRNAs are shown by color codes presented on the right side of the panel. (D) Ratio of full length and truncated miRNA matching reads. The columns show the percent of full-length (fl), 5’-truncated (5’tr) and 3’-truncated (3’tr) miRNAs in the input and p19-IP of p19syn and wild type plants. Values above the columns indicate the normalized read count ratio of p19-IP/input for each miRNA in a log2 scale.

Analysis of p19-bound sRNAs from mock-inoculated p19syn plants revealed that p19 binds efficiently endogenous sRNAs (Fig 2A and 2B), including members of several miRNA families (S3A Fig) [73]. Surprisingly, the analysis of p19-bound sRNA libraries derived from both CymRSV-infected wt *N*. *benthamiana* (“cis“-p19) and Cym19stop-infected p19syn plants (“trans-p19“) have shown a different picture: p19 bound almost exclusively vsiRNAs but not endogenous sRNAs (Fig 2A and 2C–2F). This suggests that the abundantly produced vsiRNAs may outcompete the plant sRNAs from p19 binding during virus infection. Specific enrichment of vsiRNAs versus endogenous miR159, one of the most abundant miRNA, was quantified by Northern blot analysis. p19 had a much weaker affinity for miR159 during virus infection: the IP/input ratio of p19 bound miR159 was 1.2 mock-inoculated samples, while during virus infection (Cym19stop-infected p19syn plants) it dropped to 0.29 (Fig 3A). We also quantified the percentage of enrichments in case of vsiRNAs and endogenous sRNAs within p19 IPs compared to inputs from our deep seq data (Fig 2A). The input library of Cym19stop-infected p19syn plants contained 28% *N*. *benthamiana* reads while in the p19-IP they represented only 2% (p19 specifically enriched vsiRNAs from 72% in the input to 98% in the p19-IP). Similarly, in the CymRSV-infected wt plant 12% *N*. *benthamiana* reads in the input sample has dropped to 1% (p19 enriched the 88% vsiRNAs of the input to 99% in the p19-IP). We concluded therefore that p19 ability to sequester endogenous sRNAs is strongly decreased by the virus infection and p19 preference to vsiRNAs does not depend on the expressional origin of p19 protein (viral vs transgenic expression).

To better understand the biological relevance of vsiRNA-mediated endogenous sRNA binding and out-competition/release from p19 sequestration we analyzed the behavior of known miRNA-target mRNA pairs [73]. We compared RNAseq data obtained from mock-inoculated p19syn plant samples (when p19 binds to miRNAs) and from Cym19stop virus-infected p19syn plant samples (when p19 binds preferentially vsiRNAs while miRNAs are outcompeted/released). In the absence of the virus, p19 efficiently bound miRNA duplexes (S3A Fig) and this correlated with elevation of most of the miRNA-target mRNAs as the consequence of miRNA duplex sequestration by p19 and inability to program miRISC for cleavage (p19syn compared to wt *N*. *benthamiana*, S3B Fig). Upon Cym19stop virus infection however, the levels of most miRNA target RNAs were downregulated (compared to mock-infected p19syn) as the consequence of miRNA out-competition/release from p19 (S3B Fig). We went further and specifically looked to accumulation of trans-acting RNAs derived from TAS3 precursor, the target of miR390 [74] in a Northern blot assay (S3C Fig). In p19syn plants the level of miR390 was slightly elevated while the TAS3-derived D7 tasiRNA dropped below the detection level (compared to wt *N*. *benthamiana*). This was likely the consequence of the inhibition of the cleavage of TAS3 transcripts by p19-captured miR390. Indeed, miR390 is efficiently enriched in p19 IP (p19syn mock-infection, S3A Fig). When p19syn plants were infected with Cym19stop virus, miR390 binding by p19 decreased (S3A Fig), and consequently the activity of miR390 was restored that lead to D7 tasiRNA accumulation (to a similar level as detected in wt *N*. *benthamiana*, S3C Fig). Altogether our findings support the hypothesis that during virus infection p19 preferentially binds vsiRNAs while endogenous sRNAs are outcompeted/released from binding.

### p19 preferentially binds perfect sRNAs duplexes without 5’-nt sorting

High-throughput sequencing analysis showed that CymRSV-derived vsiRNAs produced during infection have a strong bias towards positive strand polarity (95% positive, 5% negative polarity) (Fig 3B and S4 Fig). These data, which are in line with our and other previous observations, suggest that the majority of vsiRNAs are produced from fold-back structures of the positive strand of the viral RNAs [27,30,31,75]. Hot spots of vsiRNA generation were observed (S4 Fig) as earlier [31,75].

The polarity analysis of p19-immunoprecipitated vsiRNAs revealed a more equilibrated positive/negative strand ratio (significant enrichment in negative strand derived vsiRNAs with 65% positive, 35% negative strands) in the CymRSV-infected plants (Fig 3B). In Cym19stop-infected p19syn plants 79% of vsiRNAs produced were positive-stranded (21% negative), while p19syn—immunoprecipitated the ratio changed to 62% positive, 38% negative (Fig 3B). Based on these we conclude that p19 preferentially enriches positive-negative ds-vsiRNA pairs possessing perfect duplex structure.

**Fig 4:**
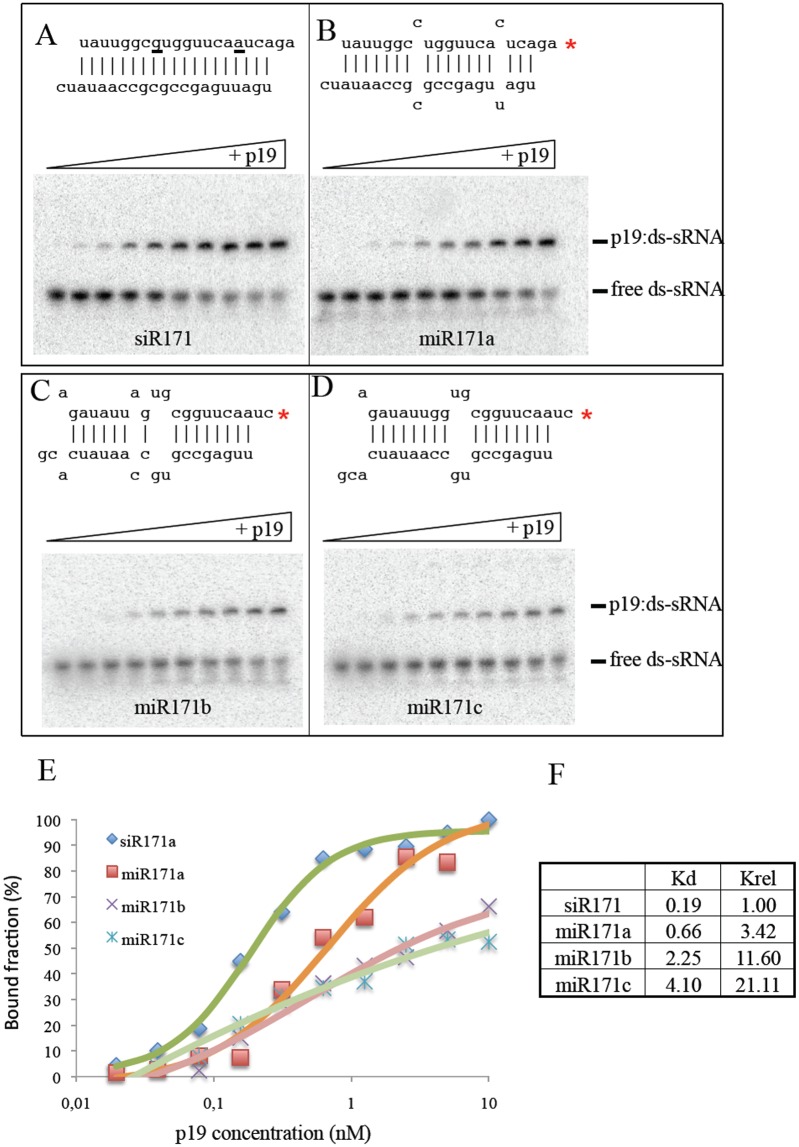
Affinity of p19 to perfect or mismatch-containing duplex si/miRNAs *in vitro*. Band shift assay of perfect duplex siR171 (A), and mismatched miR171a (B), miR171b (C) and miR171c (D) duplex RNAs’ with p19 protein. The structure of dsRNAs is shown above the gel pictures. Direct measurement of the absolute apparent dissociation constant Kd values (F) were calculated as previously described [57] based on the quantification of band intensities of p19:dsRNA bound fraction as a function of p19 protein concentration (E). Relative dissociation constant (Krel) was calculated by normalization of Kd values to Kd of siR171 (F).

To formally test the impact of mismatches within the duplex sRNAs on the affinity of p19 we compared the affinity of p19 protein towards the miR171 duplex miRNA family (*Arabidopsis* miR171a, miR171b, miR171c all containing mismatches) and a perfect artificial siR171 duplex (for structures see Fig 4A–4D) using *in vitro* electro-mobility shift assay [57]. The presence of mismatches within the stem of sRNAs strongly reduced p19 binding affinity towards duplex sRNAs (Fig 4A–4F). Consistent with our findings, it has been also shown previously that p19 preferentially binds to perfect sRNAs duplexes but not imperfect miRNAs duplexes [76].

We have also analyzed the 5’-nucleotide preference of p19 binding. No 5’-nucleotide sorting of vsiRNAs in the p19 complex was observed regardless of p19 expressional context (from the virus or the transgene) (Fig 2C and 2E). The relative abundance of sRNAs possessing different 5’-nucleotides closely followed the ratio of the input samples. The distribution of p19 bound vsiRNAs along the viral genome was found to be similar to that in the input (S4 Fig) showing that there is no sequence preference in p19 vsiRNA binding.

### p19 induces 3’ shortening of sequestered sRNAs

To better understand the p19 protein effects on vsiRNAs we analyzed the size distribution of these during infection. Upon CymRSV infection vsiRNAs produced are predominantly of 21nt and 22nt in length (Fig 3C). In addition to these, the 20nt long vsiRNAs are still present although at much lower level. In Cym19stop virus-infected wt plants we observed a shift towards slightly longer forms: most of the vsiRNAs were 22nt long, the abundance of the 21nt and 20nt long forms being reduced (Fig 3C). These results are in line with our previous findings [31]. miRNAs having enhanced electrophoretic mobility were also detected earlier in the presence of p19 [62,77].

To test if shortening is indeed an effect of p19 protein itself we analyzed parental virus- (Cym19stop) derived vsiRNA when p19 was provided “in trans“. The length shift to 1- or 2-nucleotide shorter vsiRNA forms was confirmed (Fig 3A–3C). Shortening of endogenous miRNAs was also observed in the absence of virus infection (Fig 3D). Analysis of selected endogenous miRNAs, where the precise sequence and biogenesis/maturation are known, allowed us to establish that the truncation occurred at the 3’ end but not 5’ end. The truncation of miRNAs happened mainly in p19-sRNA complexes as was observed in p19-IP, however not all p19 bound miRNAs are truncated and the reason for this has not been clarified yet (Fig 3D). Nuclease treatments on *in vitro* bound p19:siRNA complexes further confirmed that the p19 protein can protect the double-stranded stretch of sRNA duplexes (S5 Fig). The exonuclease (RNaseA)-mediated digestion occurred in discrete 1- and 2-nucleotide steps while the dsRNA region (19nt length) was protected by the p19 protein. Shortening of sequestered sRNAs, therefore, is not dependent on the virus infection, occurs on 3’ end, involves both vsiRNAs and miRNAs and is likely the direct consequence of p19-binding and exonuclease activity.

### Differential effect of p19 on vsiRNAs loading into AGO1 and AGO2

Multiple AGOs were shown to have antiviral functions. In *A*. *thaliana* and *N*. *benthamiana* AGO1 and AGO2 were described as the most important effectors while others such as AGO5, 7 and 10 to have minor roles during antiviral silencing [13,34,36,44]. The current model of the inhibitory effect of p19 suggests that sequestering vsiRNAs prevents AGO loading. The inhibitory effect of p19 protein on RNA silencing during infection was quite evident. In fact, wt virus infection showed strong viral symptoms that culminated in complete necrosis and collapse of the plants while the Cym19stop-infected wt plants recovered from viral infection [70], the virus accumulation was restricted to the vascular tissues and a few cell layers around the veins ([70] and S2 Fig).

**Fig 5:**
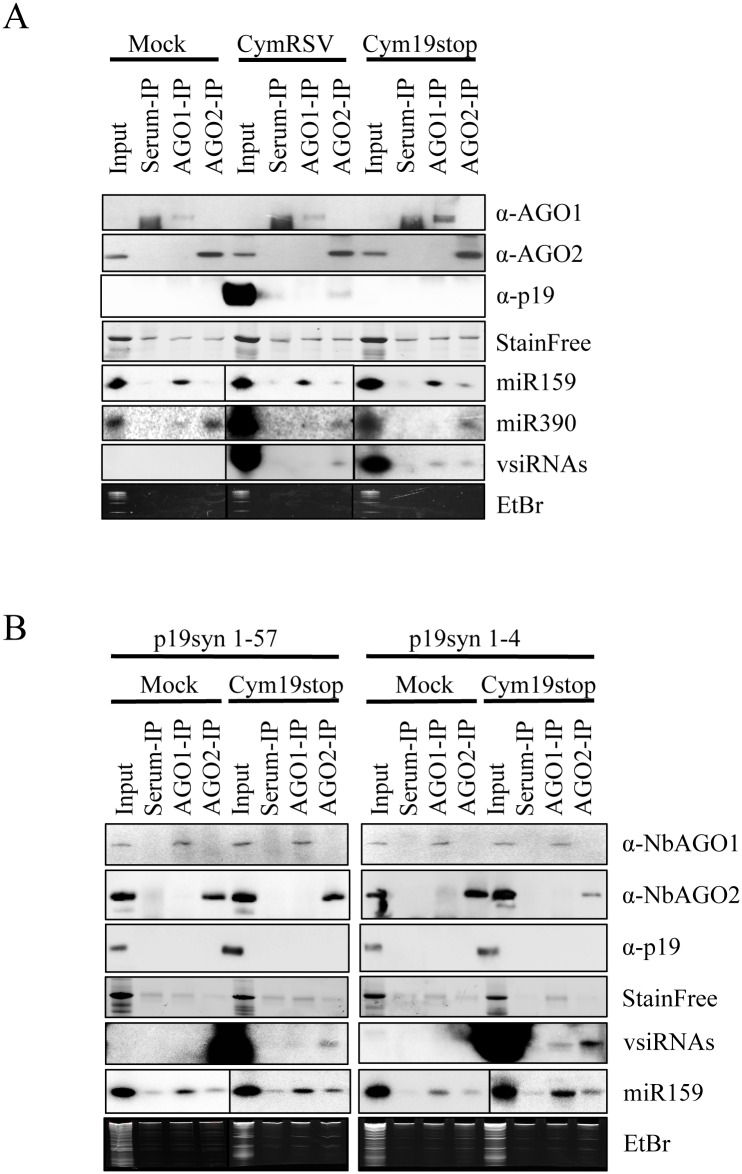
Northern blot analysis of NbAGO1- and NbAGO2-bound vsiRNAs and miRNAs. (**A**) Serum- NbAGO1- and NbAGO2-immunoprecipitations from mock-, CymRSV- or Cym19stop-infected wt *N*. *benthamiana* plants as shown: upper panels, Western blots for AGO1, AGO2 and p19 are shown (StainFree gel picture shown as loading control); lower panels, Northern blots of AGO1-specific miR159, AGO2-specific miR390 and vsiRNAs (ethidium-bromide stain shown as loading control). (B) Serum- NbAGO1- and NbAGO2-immunoprecipitations from mock- or Cym19stop-infected transgenic p19syn lines as shown: upper panels, Western blots for AGO1, AGO2 and p19 (StainFree gel picture shown as loading control); lower panels, Northern blots of AGO1-specific vsiRNAs and miR159 (ethidium-bromide stain shown as loading control).

**Fig 6:**
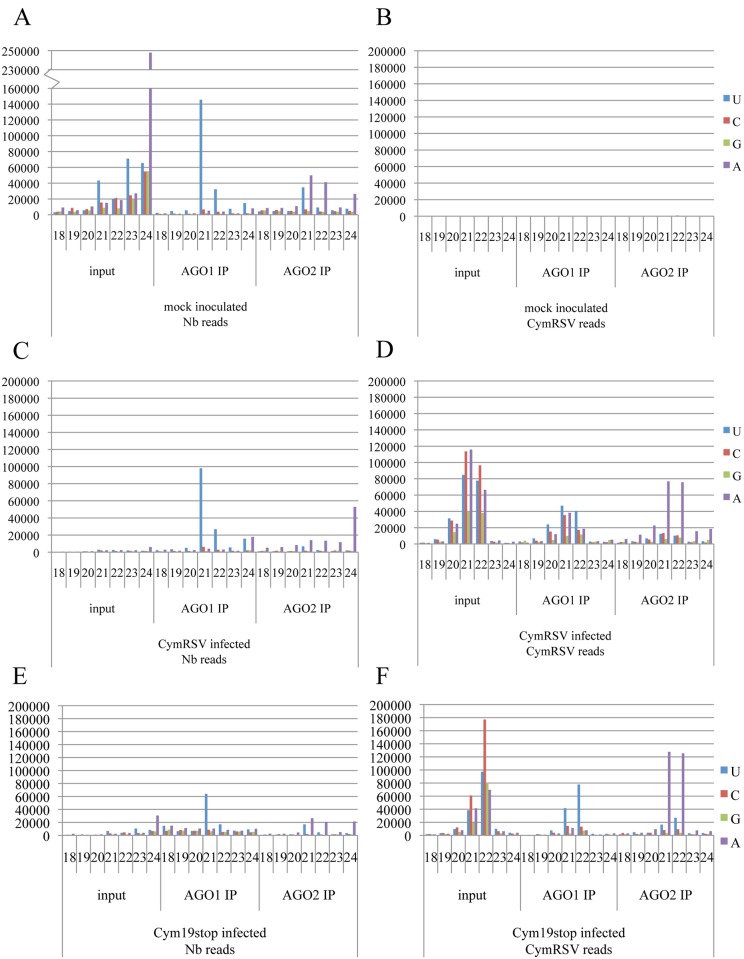
Deep sequencing analysis of AGO1- and AGO2-bound sRNAs. *N*. *benthamiana* specific reads from mock-inoculated (A), CymRSV- (C) and Cym19stop-infected wild-type plants (E). The vsiRNA reads of the same samples are presented in (B), (D) and (F) respectively. 5’ nucleotides of vsiRNAs are indicated by color code on the right. Size classes (nucleotide) of sRNAs are indicated by numbers. Read counts were normalized to 10^6^ total reads. Note the different scale in panel A.

To get a better insight into the detailed mechanism of p19 actions we analyzed the AGO1- and AGO2-bound vsiRNAs in CymRSV- and Cym19stop-infected wt *N*. *benthamiana* by co-immunoprecipitations (Fig 5) followed by deep sequencing analysis (Fig 6 and S6–S10 Figs). Loading of siRNAs into a particular AGO is preferentially directed by their 5’-terminal nucleotide: AGO1 prefers sRNAs having 5’U while AGO2 preferentially binds 5’A sRNAs [78,79]. As expected, the AGO1 co-immunoprecipitated plant sRNAs possessed predominantly 5’U while AGO2 immunoprecipitated sRNAs mainly 5’A, with a relatively high amount of 5’U species (Fig 6). We have also found 5’U endogenous sRNA binding by AGO2 when we processed the raw data obtained from previous report [36].

We expected that p19 would drastically reduce the loading of vsiRNAs into AGO1 and AGO2. Surprisingly, vsiRNA loading into AGO1 was compromised in the presence of p19 (during CymRSV-infection compared to Cym19stop-infection): we observed relatively high “background” of vsiRNAs without 5’ sorting preference in AGO1 (compare Fig 6D with 6F). Conversely, the amount of vsiRNAs and their 5’ sorting into AGO2 was very similar during CymRSV- and Cym19stop- infections (Fig 6D–6F). This suggests that the presence of p19 preferentially impact vsiRNAs’ AGO1 but not AGO2 effector loading. In the same time (the same sample set) endogenous sRNAs were efficiently precipitated as 5'U by AGO1-IP proving that the IP worked correctly (Fig 6C–6E). Note that the reads of endogenous sRNAs in CymRSV and Cym19stop are lower compared to mock-infected sample due to the high amount of vsiRNA presence (that impacts the bias during deep sequencing).

The AGO1 IP derived from CymRSV-infected plants contained a similar miRNA profile as the mock inoculated plants, in contrast to AGO2 IP in which the levels of analyzed miRNAs were reduced (Fig 6C and 6D and S6 and S7 Figs). Importantly, this occurred only in wt CymRSV infection when a high level of p19 is expressed. Efficient incorporation of vsiRNAs into AGO2 but not AGO1 may cause the out-titration of AGO2-bound endogenous sRNA species (during CymRSV infection). In contrast, during Cym19stop-infection AGO1-loading occurred as expected predominantly by 5’U-sorting of vsiRNA and endogenous sRNAs (Fig 6E and 6F). The obtained results were confirmed with a second AGO1 and AGO2 IP that gave very similar results although had slightly higher background of contaminating 24nt species (S9 Fig). These findings suggest that p19 protein itself compromises AGO1- but not AGO2-loading during viral infection.

The specific impact of p19 on vsiRNA AGO1-loading found in the deep sequencing analysis was also confirmed by Northern blot analysis. vsiRNAs loading into AGO1 was less efficient than AGO2 in CymRSV-infected plants when the p19 was provided in “cis” (Fig 5A) or in “trans” when two independent p19syn lines were infected with Cym19stop virus (Fig 5B).

We also analyzed the distribution of AGO1- and AGO2-bound vsiRNA along the viral genome. This generally followed the biogenesis of vsiRNAs and we could not define any sequence preference or specific hotspots of AGO1- or AGO2-loading (S8 Fig). The strong spikes of certain vsiRNAs may arise due to the sequencing bias, therefore, do not necessarily represent vsiRNAs preferred for binding [80].

## Discussion

Most of the identified VSRs are multifunctional, and this nature of VSRs often causes serious difficulties in the exploration of their action during the natural virus infection. The inactivation of VSRs often leads to loss of viability of the given virus, due to their multifunctional nature. It is hard therefore to separate the suppressor’s impact and virus infection effects. Either transgenic or transient expression of VSRs without the parental virus background might not reflect the natural interaction with the host cellular machinery. As a consequence of these difficulties, the molecular mechanisms of the action of VSRs and their impact on the host often remain elusive. To overcome this pitfall, we combined the transgenic expression of the VSR (p19) suppressor with its authentic suppressor mutant virus (Cym19stop)-infection in a novel experimental setup.

### Features of ds-sRNAs bound by p19

During virus infection, high amounts of vsiRNAs are produced. These vsiRNAs are efficiently sequestered by p19 suppressor inhibiting their incorporation into RISC [67]. The consequence of p19 vsiRNA binding is that the strong positive strand bias of vsiRNA biogenesis in the input sample (95:5 positive/negative) is changed to a more equilibrated positive/negative stand ratio (65:35 positive/negative). This result suggests that there are qualitative structural differences between vsiRNAs and that p19 preferentially binds vsiRNAs derived from perfect dsRNA or highly structured RNA species. The preference of p19 towards even structured ds-vsiRNAs is in agreement with p19 crystal structure: p19 homodimer leans on the ds-sRNA backbone. If the backbone structure is distorted by mismatches, the sRNA could become less accessible to p19 sequestration. Indeed, p19 bound siR171a perfect duplex with higher affinity than natural miR171a, miR171b or miR171c duplexes (Fig 4). This may likely be one of the reasons why ds-vsiRNAs are preferred by p19 instead of mismatches containing endogenous miRNA duplexes during viral infection (Figs 2 and 3A). The excess of vsiRNAs over endogenous miRNAs in virus-infected plant may also contribute to the preferential binding of vsiRNAs by p19. Moreover, the difference in the biogenesis of miRNAs versus vsiRNAs could also be a further important factor in the mechanism of the sRNA sequestration by p19.

In addition to the previous findings, we have also shown that sRNA binding by p19 happens without 5’-end nucleotide selection including vsiRNAs or endogenous miRNAs.

### Shortening of p19-bound sRNAs

Previous studies [62,77] have reported the truncation of p19-bound sRNAs by 1 or 2 nucleotides. In the case of vsiRNAs, the site of truncation (5’ vs. 3’ end) cannot be defined since the generated vsiRNAs started from almost every single nucleotide of viral genome (S4 and S8 Figs). During Cym19stop-infection on wild-type plants truncation does not occur while in p19syn plants, which provide p19 in *trans*, it can be observed. Shortening also happens on miRNAs in p19syn plants without virus infection. In summary, the truncation is likely induced directly by p19-binding and is not due to the virus infection or restricted to a specific class of sRNAs. Why shortening does not happen in the absence of p19, on the free vsiRNAs, which theoretically would be more accessible? In cells endogenous free sRNA duplexes (e.g., miR168/miR168star) [54] and free vsiRNAs in Cym19stop infection can be observed [32,67]. The stability of these sRNAs (miRNAs and vsiRNAs) is conferred by HEN1-mediated methylation [11]. The crystal structure of p19:siRNA complex shows that the last two single-stranded nucleotides at 3’ terminus of siRNAs are protruding from the complex [57,58]. Furthermore, the p19 bound vsiRNAs are not methylated at 3’ terminus [65] therefore may be sensitive to exonucleases. We propose therefore that p19 binding inhibits sRNAs methylation and as a consequence of this the protruding unprotected two nucleotides at the 3’-end of sRNAs are trimmed by cellular 3’-exonucleases. Whether the truncation of sRNAs is a simple byproduct of binding or has a definite biological importance remains to be seen. Trimming of sRNAs may inactivate and render them incompetent for AGO-loading. Contradictory to this, we find efficient binding of 19nt and 20nt vsiRNAs by AGO2 (Fig 6D, S9D Fig). This observation supports the “catch and release” of vsiRNAs by p19 proposed earlier [81].

### The effect of p19 on viral symptom development

It has been long suggested that VSRs interfere with endogenous silencing pathways, and this may contribute to the viral symptom development [1,61–64]. Constitutive expression of p19 in *N*. *benthamiana* leads to the development of a strong phenotype that is quite different from symptoms observed during parental viral infection (Fig 1 and S2 Fig). The strong phenotype of p19syn plants may arise, at least in part, due to the sequestration of endogenous sRNAs by p19. Indeed, we could immunoprecipitate endogenous miRNAs with p19 from transgenic plants (Fig 2B). Importantly, however, miRNA sequestration by p19 provided either *in cis* or *in trans* was drastically reduced when the virus was present (Fig 2C–2F). Importantly, miRNA out-competition/release correlated with downregulation of miRNA targets (S3B Fig) and reestablishment of tasiRNA biogenesis (S3C Fig). Out-competition/release of p19-bound endogenous sRNAs/miRNAs upon virus infection seems to be biologically relevant and could have an important role in moderating the virus impact on plant. This needs to be further investigated. In conclusion therefore, our findings deny the model in which miRNA binding by p19 is the key step for the development of virus-induced symptoms [1,61–64]. It is more likely that p19 has an indirect effect through the specific inhibition of antiviral plant response and the viral symptoms are the outcome of a complex virus-host interaction during the viral invasion of plant cells.

### Loading vsiRNAs into AGOs

What could be the criteria for vsiRNA selection by AGO-loading machinery? We previously observed that in wt tombusvirus infection, p19 protein prevents vsiRNA loading to AGO/RISC complexes, however, even in the absence of p19 only a small fraction of the vsiRNAs is loaded into effector complexes (Fig 5) the rest remaining in a free, probably double-stranded form [32,67]. This suggests that a big part of the abundantly produced vsiRNAs is AGO-incompetent, or there is no free AGO protein present to be loaded into. The structure of the ds-sRNA stem could be an important feature for vsiRNA selection into AGOs (as we have shown for p19). A similar analysis of sRNA duplexes as in the case of p19 cannot be done, since in p19 binding both strands of ds-sRNAs are retained, while in AGOs, after loading, one strand is eliminated. The other possibility of the inability of vsiRNAs to load into effector complexes could be the shortage of silencing proteins like DCL/DRB or AGOs during the assembly of these effectors. It was shown that specific regulatory mechanisms are induced by the virus to dampen silencing: translation of AGO1 protein is decreased by the suppressor-mediated miR168 over-accumulation [54,66]. However, the down-regulation of AGO1 protein was not observed in p19syn plants (Fig 1B). The reason for this could be the relatively low level of p19 produced from transgene compared to virus infection (Fig 5).

Another important observation is that vsiRNAs loading is selectively prevented mainly into AGO1 but not AGO2 in the presence of p19 in both CymRSV infected wt or Cym19stop infected p19syn plants (Fig 5). During virus infection, the decrease in the translation of AGO1 protein leads to accumulation of AGO2, due to the absence of AGO1-miR403-mediated posttranscriptional down-regulation of AGO2 [54,66]. One possibility, therefore, is that the vsiRNAs will be loaded into the available AGO2 while AGO1-loading will be decreased. We could not observe a significant increase of AGO2 protein in the presence of p19 (Fig 1B). The other possibility is that AGO1 and p19 compete for the same set of vsiRNAs while AGO2 requirement for vsiRNA features is different. p19 therefore, would selectively impact AGO1 but not AGO2-loading.

Interestingly, we observed relatively high p19-depending “background” of vsiRNAs without 5’ sorting in AGO1 IP, unlikely to be AGO1-incorporated sRNAs. p19 could affect the connection between biogenesis/loading complexes DCLs/DRBs with AGO1 effector [7,9]. In line with this hypothesis, it was demonstrated that p19 can compromise the transfer of siRNA from DICER-R2D2 into RISC complex using Drosophila embryo extracts based *in vitro* system [59].

Regardless of the reason of how AGO1-loading is compromised by p19, it seems that AGO2 is not enough to fight off the virus and help the plant to recover in the absence of AGO1-loading/activity. It has been suggested previously that AGO2 but not AGO1 plays role in the antiviral response against tombusvirus infections, including *Tomato bushy stunt virus* (TBSV) [47,48]. We have done TBSV-VIGS (Virus Induced Gene Silencing) experiment, using p19 inactivated virus vector (TBSVp19stop), which carried Nb-PDS and Nb-AGO1 sequence (S11 Fig). When NbAGO1 was silenced by VIGS the virus accumulated at higher level and plants have shown stronger phenotype (S11 Fig). The obtained results further support the idea that AGO1 has a major role in antiviral response against tombusvirus infection. However, the additional role of other plant AGOs in antiviral response remained to be explored and it likely depends on specific features of the highly diverse plant viruses. The availability of CRISPR/Cas9 system for plant research will also help to clarify the specific roles of plant effectors in antiviral silencing response.

## Materials and Methods

### Design and construction of the synthetic CymRSV p19 gene

The synthetic CymRSV ORF5 (p19) was essentially constructed following the previously described antivirus-induced transgene silencing strategy [82]. As the first step, we introduced, in CymRSV ORF5, all possible silent point mutations by selecting those most compatible with the *N*. *tabacum* codon usage. The resulting nucleotide sequence was further modified to avoid the presence of cryptic splicing and polyadenylation signals using Net2gene splicing prediction (http://www.cbs.dtu.dk/services/NetGene2/) and HCpolyA (http://bioinfo4.itb.cnr.it/~webgene/wwwHC_polya_ex.html) software, respectively. The synthetic ORF5 (S1A Fig) was synthesized by Life Technologies and cloned in pJIT61 [83] between the CaMV 35S promoter and 35S terminator. The gene cassette was excised with *Kpn*I and *Bgl*II and cloned in *Kpn*I-*Bam*HI of pBin19 (pBinCymRSVp19syn)

### Transgenic plants

*N*. *benthamiana* was transformed with the recombinant *Agrobacterium tumefaciens* strain C58C1 (pGV2260) harboring the plasmid pBinCymRSVp19syn, and kanamycin-resistant plants were regenerated as previously described [83]. The primary transformants were checked for the presence of *p19syn* transgene by PCR and for the expression of the p19 protein by Western blotting with the anti-CymRSV-p19 antibody as previously described [56].

### Plant agroinfiltration

*N*. *benthamiana* plants were grown at 22°C. At six-leaves stage plants were infiltrated with *A*. *tumefaciens* C58C1 harboring the appropriate constructs in the pBIN61 plasmid. pBIN61-Cymp19 and pBIN61-GFP were grown on selective media overnight, resuspended in the infiltration buffer (10 mM MES, 0.15 mM acetosyringone, 10 mM MgCl_2_) kept on 25°C for 4h, and subsequently infiltrated into wild-type or p19syn plant leaves at OD_600_ = 0.4.

### Virus infections

*In vitro* transcription of CymRSV, Cym19stop, TBSV-PDS-GFP and TBSV-PDS-AGO1-1 RNAs from linearized template plasmids and inoculation of RNA transcripts onto *N*. *benthamiana* plants were performed as described previously [84]. CMV Y-sat infection was performed as described earlier [69].

### RNA extraction and northern blotting

Total RNA was extracted from 100 mg of leaf tissue. The homogenized plant materials were resuspended in 600 μl of extraction buffer (0.1 M glycine-NaOH, pH 9.0, 100 mM NaCl, 10 mM EDTA, 2% SDS) and mixed with an equal volume of phenol. The aqueous phase was treated with equal volumes of phenol-chloroform and chloroform, precipitated with ethanol, and finally resuspended in sterile water. RNA gel blot analysis of higher molecular weight RNAs was performed as described previously [84].

RNA gel blot analysis of 21–24 nt RNAs was performed as follows. Approximately 5 μg of total RNA was separated by 15% PAGE with 8.6 M urea and 1xTris-borate-EDTA. RNA was electro-blotted onto Hybond-NX membranes and fixed by chemical crosslinking at 60°C for 1 hr [85]. Small RNA Northern blot hybridization and analysis were performed using complementary locked nucleic acid (LNA) oligonucleotides (Exiqon, http://www.exiqon.com).

### Protein extract preparation and western blotting

Mock- or virus-infected systemical leaf tissues were homogenized in extraction buffer (150 mM Tris-HCl, pH 7.5, 6 M urea, 2% SDS, and 5% μ-mercaptoethanol). Samples were boiled, and cell debris was removed by centrifugation at 18,000 x *g* at 4°C for 10 min. The supernatants were resolved on 12% SDS-PAGE, transferred to Hybond PVDF membranes (GE Healthcare) and subjected to Western blot analysis. For detection anti-p19 [70], NbAGO1 [86] and NbAGO2 custom antibody were used. NbAGO2 antibody was generated by immunization of rabbits with the synthetic peptide (CLEDPEGKDPPRDVF)(GenScript, http://www.genscript.com/). The proteins were visualized by chemiluminescence (ECL kit; GE Healthcare) according to the manufacturer’s instructions.

### Immunoprecipitations of p19, NbAGO1 and NbAGO2

For immunoprecipitation, 1–5 grams of mock-, CymRSV- or Cym19stop-infected *N*. *benthamiana* leaves showing systemic symptoms (or leaves at the same stage and positions from mock-inoculated plants) were collected, ground in 1:3 (w/v) amount of immunoprecipitation buffer (40 mM HEPES/KOH 7.4, 100 mM KOAc, 5 mM MgOAc, 5% glycerol, freshly added 4 mM DTT), and cleared by centrifugation (twice at 15,000 x *g* for 5 min). Cleared lysates were kept on ice until immunoprecipitation with antibody-coated protein A-Sepharose (GE Healthcare). Beads were washed before adding the antibodies (described earlier). For mock immunoprecipitation preimmune serum was used. Antibodies coated beads were incubated with the relevant cleared lysates for 4h at 4°C. After immunoprecipitations the beads were washed five times with ice-cold immunoprecipitation buffer for 2 min each. Input extracts and eluates of immunoprecipitations were used for Western and Northern blot analysis.

### Library constructions for high-throughput sequencing

The library preparation was described previously [31], shortly: RNA samples were purified by cutting the sRNA region from 8% denaturing polyacrylamide gels (acrylamide:bisacrylamide (19:1) 1xTBE, 8.6 M urea). After gel electrophoresis, the gels were stained by SYBRGold (Thermo Fisher Scientific). Bands at the small RNA range were cut out and crushed. Gel particles were shaken overnight in RNase free water at 4°C, followed by RNA isolation (described above). TruSeq Small RNA Sample preparation kit (Illumina) was used for library preparation; we followed the manufacturer’s protocol. In the case of the AGO1- and AGO2- immunoprecipitation 9 libraries were pooled together. In the case of the p19 immunoprecipitations, 4–4 libraries were pooled together. The libraries were sequenced on Illumina HiScanSQ platform (UD-GenomMed Medical Genomic Technologies Ltd., Debrecen, Hungary) that yielded approximately 100 M reads per lane (50bp, single end) (S10 Fig).

RNA-seq library preparation was done according to the manufacturer protocol (TruSeqStranded mRNA Library Prep Kit). The libraries were sequenced on Illumina HiScanSQ. 135 M 100 bp paired-end reads were produced per lane. 3 samples were pooled together on a lane. The libraries were submitted to GEO and can be accessed through series accession number GSE77070.

### Preparation of synthetic siRNA for RNaseA assay

One hundred nanograms of synthetic sRNA (5’-UGAUUGAGCCGCGCCAAUAUC-3’) was 5’ end labeled by T4 polynucleotide kinase (Fermentas) with ^32^P isotope. After the reaction was stopped, 10 ng was saved for further process and the rest was mixed with 500 ng of unlabeled synthetic RNA with the sequence of 5’-UAUUGGCGCGGCUCAAUCAGA-3’. The mixture was heated to 95°C for 2 minutes in a thermocycler and was cooled to 5°C (2°/2 min) to gain 19 nt perfectly matched double strand with 2 nt protruding at the 3’ end. The sufficient amount of DNA loading dye was added to the mixture and to the saved labeled single stranded RNA. Both samples were run on a 8% acrylamide:bis-acrylamide 19:1 1xTBE gel. Gel was directly exposed. The dsRNA region was cut from the gel. The gel piece was shredded using a 0.5 ml tube with several holes in the bottom in a 2 ml tube via centrifugation. The shredded gel pieces were shaken overnight in 500 μl of 300 mM NaCl at 4°C. Gel pieces were filtered out by using Spin-X column (Corning). 400 μl of dsRNA solution was precipitated by adding 20 μg glycogen (Fermentas) and 1 ml ethanol. The precipitated ds-RNA was resolved in IP buffer described before.

### RNase A assay

Purified p19 described earlier was used for the assay [56]. A dilution series of p19 (~1 μg) was made in 1x IP buffer. An equal amount of gel-purified ds-siRNA was added to each p19 dilution and incubated at room temperature for 10 min. Then 10 ng of RNase-A (Sigma) was added and incubated for 10 and 30 min at room temperature. After incubation the samples were mixed with DNA dye and ran on a 16% acrylamide: bis-acrylamide (19:1) 1xTBE gel. Decade marker (Ambion) and synthetic RNAs were used as size markers. Gels were dried and were directly exposed.

### Electrophoretic mobility shift assays

For band shift assays wild-type p19 protein was purified from *E*. *coli* as described previously [56,67]. Custom RNAs used were ordered from Dharmacon, (http://dharmacon.gelifesciences.com) for sequence see Fig 4. Labeling and annealing of si/miRNA duplexes was carried out as described previously [67]. Purified p19 protein and labeled si/miRNAs were incubated for 30 min at room temperature in band-shift buffer [67]. Complexes were resolved on 8% polyacrylamide 0.5xTBE gels. Gels were dried and exposed to a storage phosphor screen and bands quantified (Molecular Dynamics Typhoon Phosphorimager, GE Heathcare).

### In situ hybridization

In situ hybridization was performed as previously described in [87]. Detection of viral RNA expression patterns were made by using nonradioactive in situ hybridization on histological sections of leaf tissues. Digoxigenin labeled antisense RNA probe was synthesized by *in vitro* transcription from the linearized CymRSV construct.

### Bioinformatic analysis of sRNA libraries

After demultiplexing of the raw data we used the UEA workbench version 3.0 [88] for adapter removal. Quality control consisted of filtering out reads with less than 14 nt (without the adapter sequences) and reads showing low complexity. We used PatMaN v1.2.2. [89] to align reads allowing 0 mismatches. Reads not matching either genome were removed. Reads passing quality control is referred as “total” in this article.

In Fig 3B reads were normalized to 1 million viral reads. In S7 Fig reads were normalized to 1 million *N*.*benthamiana* genome matching reads. In all other cases reads were normalized to 1 million total reads.

### Bioinformatic analysis of mRNA libraries

After demultiplexing we used FastQC 0.10.1 to check general attributes. Trim_galore 0.4.1 and FASTX Toolkit 0.0.13 were used to remove adaptor sequences, low quality bases, reads under 20 nt and unpaired reads. Bowtie2 [90] was used to align reads to Nbv5 [91] transcriptome database. Reads were counted for homologs of known miRNA targets. NCBI-blast+ 2.2.28 [92] was used to validate miRNA targets. Samtools 0.1.19-96b5f2294a was used during alignment evaluation. Read counts were normalised to 1 million total reads.

## Supporting Information

S1 Fig Synthetic CymRSV p19 ORF used for generation of p19-expressing transgenic *N*. *benthamiana* plants (p19syn). (A) Nucleotide sequence of CymRSV p19 synthetic ORF used for plant transformation (*p19syn*). Start and stop codons are in bold, modified nucleotides are in red lowercase letters, red and green highlights are restriction sites used for cloning. (B) Alignment of CymRSV p19 ORF with the synthetic p19 ORF (p19syn). Nucleotide sequences show 68% similarity. (C) Amino acid alignment of p19 translated from *p19syn* and CymRSV p19 ORF shows 100% identity.
(DOCX)

S2 Fig Comparison of p19syn plants’ phenotype with CymRSV- or Cym19stop-infected viral symptoms and virus distribution in wt or p19syn plants. (A) Characteristic phenotype of p19syn (1–57 line)(blue arrow) and virus-induced sytemic symptoms on wt or p19syn plants (red arrow) are shown. Pictures were taken at 10 dpi. (B) *In situ* hybridization of Cym19stop-infected wt *N*. *benthamiana* and p19syn plants or CymRSV-infected wt *N*. *benthamiana* leaf cross sections showing distribution of virus within the leaf tissue. Mock-infected wt *N*. *benthamiana* cross section shown as negative control (on the right).
(PDF)

S3 Fig p19’s miRNA binding and its consequences on downstream targets in the absence and presence of the virus. (A) Abundance of p19-bound miRNAs (listed on the left) in mock- or Cym19stop-infected p19syn plants and in CymRSV-infected wild type plants. The normalized total reads are shown on a log10 scale, values under threshold (ut). Heatmap legend is shown on the right. Read counts were normalized to 10^6^ *N*. *benthamiana* genome matching read counts. (B) Ratio of miRNA-targeted mRNAs in mock-infected p19syn plants (relative to mock-infected wt *N*. *benthamiana*) and Cym19stop-infected p19syn plants (relative to mock-infected p19syn plants). Heatmap legend is shown on the right on a log2 scale. (C) Accumulation of miR390 and TAS3-derived D7 tasiRNAs in mock-infected or Cym19stop-infected p19syn plants compared to mock-infected wt *N*. *benthamiana* plants. vsiRNAs, miR159, and nucleolar small RNA U6 are shown as controls.
(PDF)

S4 Fig Distribution of vsiRNAs along the viral genome in input and p19-immunoprecipitated samples. Location of vsiRNAs on the viral genome is presented on the x-axis, read counts are shown on the y-axis. Y-axis positive and negative values represent read counts derived from the positive or negative viral strand, respectively. 21nt and 22nt vsiRNAs are indicated in blue and red, respectively. Read counts were normalized to 10^6^ “trimmed” read counts.
(TIF)

S5 Fig Shortening of p19-bound synthetic siRNAs *in vitro*. 5’ labeled siRNAs were incubated for 10 minutes with different amout of p19 and then exposed to RNase A-digestion for 10 and 30 minutes, upper and lower panel, respectively. The protected p19-bound RNA duplexes were analyzed on 16% denaturing acrylamide gel. Size marker is shown on the left.
(TIF)

S6 Fig Abundance of selected miRNAs in the mock-, CymRSV- or Cym19stop-infected input samples. miRNAs analyzed are listed on the left. The normalized read counts values are shown on a log10 scale, values under threshold (ut). Read counts were normalized to 10^6^ *N*. *benthamiana* genome matching read counts.
(TIF)

S7 Fig Relative abundance of AGO1- and AGO2-bound miRNAs in the mock-, CymRSV- or Cym19stop-infected wild type plants. Abundance of miRNAs (listed on the left) in IP fraction were calculated relative to their inputs. The normalized values are shown on a log2 scale. Heatmap is shown on the right.
(TIF)

S8 Fig Distribution of vsiRNAs along the viral genome in input, AGO1- and AGO2-immunoprecipitations from CymRSV- and Cym19stop-infected plants. Location of vsiRNAs on the viral genome is presented on the x-axis, read counts are shown on the y-axis. Y-axis positive and negative values represent read counts derived from the positive or negative viral strand, respectively. 21nt and 22nt vsiRNAs are indicated in blue and red, as shown. vsiRNAs in AGO1-IP derived from CymRSV-infected plants is likely due to the nonspecific background as discussed in the text (black asterisk). Read counts were normalized to 10^6^ total read counts.
(TIF)

S9 Fig Deep sequencing analysis of AGO1- and AGO2-bound vsiRNAs (2^nd^ biological replicate). *N*. *benthamiana* specific reads from mock-inoculated (A), CymRSV- (C) and Cym19stop-infected wild-type plants (E). The vsiRNA reads of the same samples are presented in (B), (D) and (F), respectively. 5’ nucleotides of vsiRNAs are indicated by color code on the right. Size classes of sRNAs are indicated by numbers. Read counts were normalized to 10^6^ total reads. Note the different scale in panel A.
(TIF)

S10 Fig Quality control and processing data of deep sequencing analysis of sRNA samples from input, p19-, AGO1- and AGO2-immunoprecipitated sample libraries. For detailed data processing information, please see Materials and Methods.
(TIF)

S11 Fig NbAGO1 is involved in antiviral silencing against TBSV. (A) Genomic organization of pTBSV-PDS-GFP and pTBSV-PDS-NbAGO1-1 VIGS constructs. (B) TBSV virus accumulation in NbAGO1-silenced, GFP-silenced and mock-infected (negative control) plants. (C) GFP-silenced but not NbAGO1-silenced plants show fast recovery phenotype.
(TIFF)
