# Supplementary material for: Distinct Effects of p19 RNA Silencing Suppressor on Small RNA Mediated Pathways in Plants
Source: PLoS Pathog. 2016 Oct 6;12(10):e1005935. doi: 10.1371/journal.ppat.1005935 (PMC5053613; doi:10.1371/journal.ppat.1005935)
Supplement: S1 Fig — (A) Nucleotide sequence of CymRSV p19 synthetic ORF used for plant transformation (p19syn). Start and stop codons are in bold, modified nucleotides are in red lowercase letters, red and green highlights are restriction sites used for cloning. (B) Alignment of CymRSV p19 ORF with the synthetic p19 ORF (p19syn). Nucleotide sequences show 68% similarity. (C) Amino acid alignment of p19 translated from p19syn and CymRSV p19 ORF shows 100% identity. (DOCX) [file ppat.1005935.s001.docx]

**S1 Figure**

A

*cc***ATG**GAgaGaGCaATtCAgGGtAGTGAtGTTAGaGAgCAAGCaGAtAGcGAgTGcTGGGAcGGtGGtGGtGGtGGaACaACaTCaCCtTTtAAgtTgCCaGAtGAgAGcCCaAGcCTtCAcGAaTGGAGacTtCAcCAtAGTGAaGAaAGcGAaAAcAAGGATAAcCCatTgGGaTTtAAaGAgAGtTGGAGcTTtGGcAAgGTgGTtTTcAAaAGgTActTaAGgTAtGACGGtGCtGAgACaTCtTTaCAtAGgGCatTgGGtTCaTGGGAgAGgGAcTCtGTgAAtGATGCtGCtTCaAGgTTctTgGGaCTtAGtCAAATtGGtTGcACtTAcAGCATaaGaTTcCGtGGtACaaGaCTtACttTgTCTGGtGGtTCTGGtACatTgCAaaGatTgATaGAaATGGCtATcAGGACaAAaCGtACtATGTTgCAaCCaACtCCtAGtGAgaGaGAgGGaAATGTtTCtAGAAGgCGtCCaGAgGGaACaGAgGCtTTtAAAGAgGAgAGtGAa**TAATAA**GAATTC

B

CymRSV p19 1 **atg**gaacgagctatacaaggaagtgacgttagggaacaagctgacagtgaatgttgggat 60

||||| |||| || || || ||||| ||||| || ||||| || || || || |||||

p19syn 1 **ATG**GAgaGaGCaATtCAgGGtAGTGAtGTTAGaGAgCAAGCaGAtAGcGAgTGcTGGGAc 60

61 ggaggaggaggaggtactacttctcccttcaaacttcccgacgaaagtccgagtctccat 120

|| || || || || || || || || || || | || || || || || || || ||

61 GGtGGtGGtGGtGGaACaACaTCaCCtTTtAAgtTgCCaGAtGAgAGcCCaAGcCTtCAc 120

121 gagtggaggctacatcacagtgaggagagtgagaataaggataatccccttggtttcaag 180

|| ||||| || || || ||||| || || || || |||||||| || | || || ||

121 GAaTGGAGacTtCAcCAtAGTGAaGAaAGcGAaAAcAAGGATAAcCCatTgGGaTTtAAa 180

181 gaaagctggagtttcgggaaagttgtatttaagagatatctcagatacgacggggcagaa 240

|| || ||||| || || || || || || || || || | || || ||||| || ||

181 GAgAGtTGGAGcTTtGGcAAgGTgGTtTTcAAaAGgTActTaAGgTAtGACGGtGCtGAg 240

241 acttcattgcacagagctcttggatcttgggaaagagattcggttaacgatgccgcatct 300

|| || || || || || | || || ||||| || || || || || ||||| || ||

241 ACaTCtTTaCAtAGgGCatTgGGtTCaTGGGAgAGgGAcTCtGTgAAtGATGCtGCtTCa 300

301 agatttctcggtctcagccaaatcggatgtacctatagcattcggtttcgaggaactcgt 360

|| || | || || || ||||| || || || || ||||| | || || || || |

301 AGgTTctTgGGaCTtAGtCAAATtGGtTGcACtTAcAGCATaaGaTTcCGtGGtACaaGa 360

361 ctcaccctttcgggagggtcgggaactcttcagcgtctcattgagatggcaattaggact 420

|| || | || || || || || || | || | | || || ||||| || |||||

361 CTtACttTgTCTGGtGGtTCTGGtACatTgCAaaGatTgATaGAaATGGCtATcAGGACa 420

421 aagcgcacaatgttacagcctacccccagcgaacgtgaaggtaatgtatcaagaagacgc 480

|| || || ||||| || || || || || || | || || ||||| || ||||| ||

421 AAaCGtACtATGTTgCAaCCaACtCCtAGtGAgaGaGAgGGaAATGTtTCtAGAAGgCGt 480

481 cctgaaggcactgaagccttcaaagaagaaagcgag**tag** 519

|| || || || || || || ||||| || || || ||

481 CCaGAgGGaACaGAgGCtTTtAAAGAgGAgAGtGAa**TAA** 519

C

p19syn 1 MERAIQGSDVREQADSECWDGGGGGTTSPFKLPDESPSLHEWRLHHSEESENKDNPLGFK 60

CymRSV p19 1 MERAIQGSDVREQADSECWDGGGGGTTSPFKLPDESPSLHEWRLHHSEESENKDNPLGFK 60

Consensus MERAIQGSDVREQADSECWDGGGGGTTSPFKLPDESPSLHEWRLHHSEESENKDNPLGFK

61 ESWSFGKVVFKRYLRYDGAETSLHRALGSWERDSVNDAASRFLGLSQIGCTYSIRFRGTR 120

61 ESWSFGKVVFKRYLRYDGAETSLHRALGSWERDSVNDAASRFLGLSQIGCTYSIRFRGTR 120

ESWSFGKVVFKRYLRYDGAETSLHRALGSWERDSVNDAASRFLGLSQIGCTYSIRFRGTR

121 LTLSGGSGTLQRLIEMAIRTKRTMLQPTPSEREGNVSRRRPEGTEAFKEESE 172

121 LTLSGGSGTLQRLIEMAIRTKRTMLQPTPSEREGNVSRRRPEGTEAFKEESE 172

LTLSGGSGTLQRLIEMAIRTKRTMLQPTPSEREGNVSRRRPEGTEAFKEESE
