# Supplementary figures and images for: Distinct Effects of p19 RNA Silencing Suppressor on Small RNA Mediated Pathways in Plants
Source: PLoS Pathog. 2016 Oct 6;12(10):e1005935. doi: 10.1371/journal.ppat.1005935 (PMC5053613; doi:10.1371/journal.ppat.1005935)

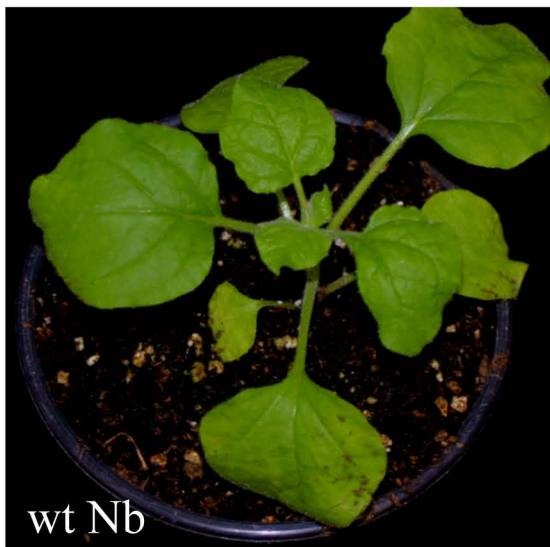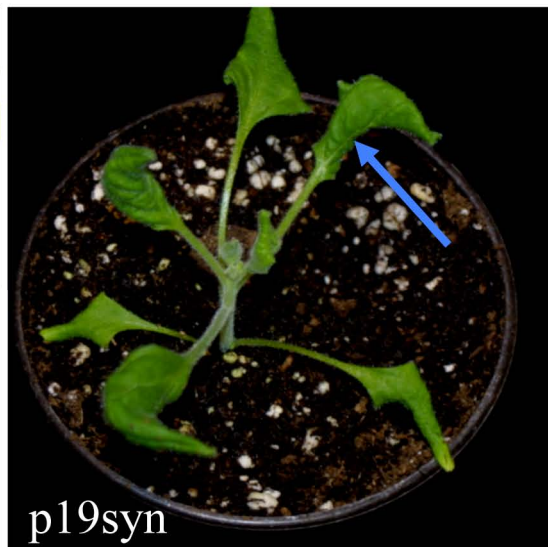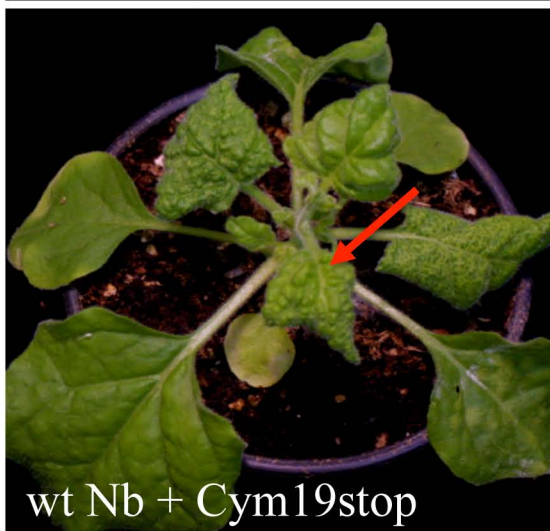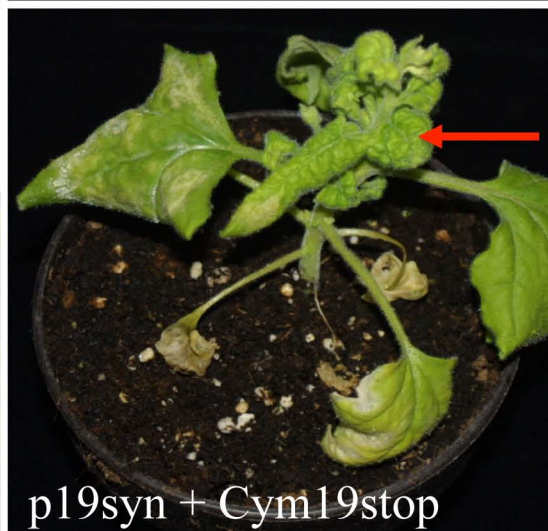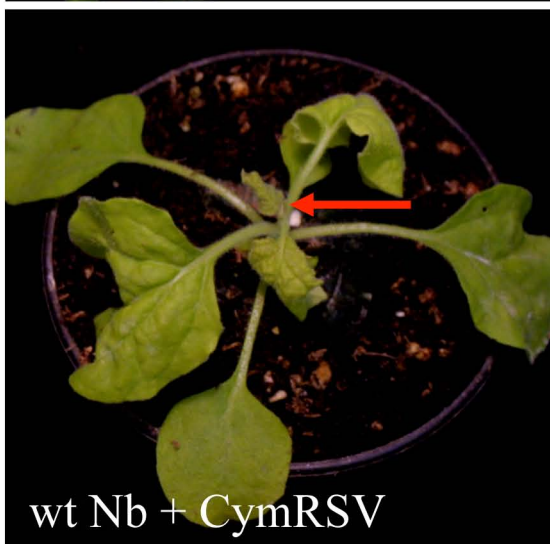

S2B

wt Nb + Cym19stop 21°C

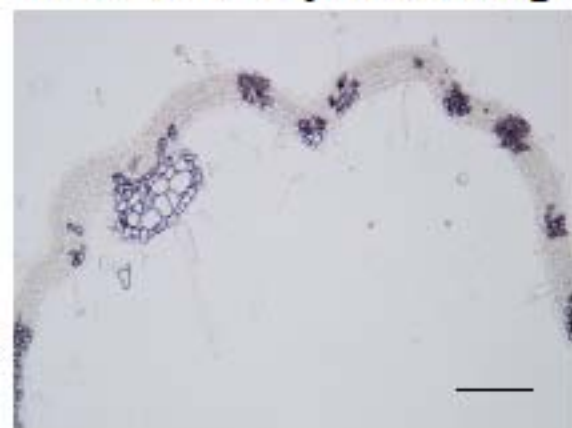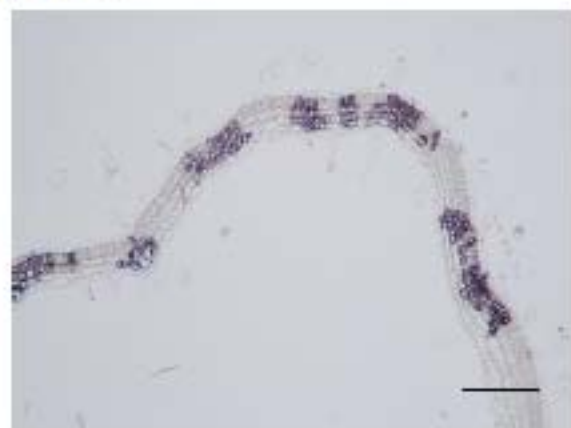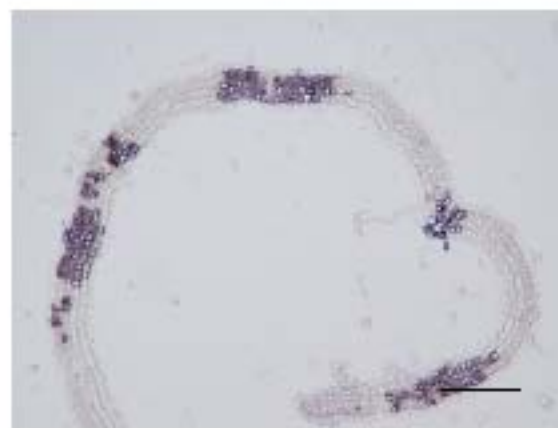

wt Nb + Mock

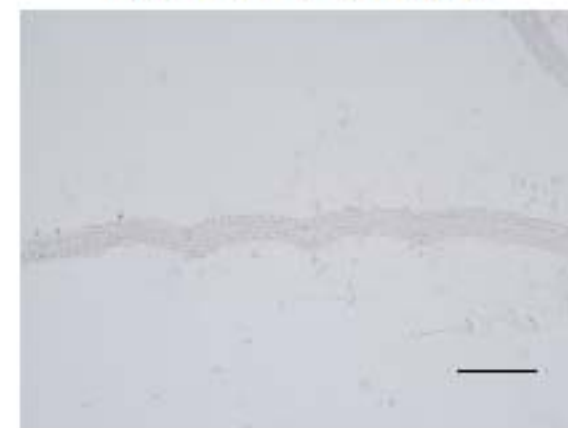

p19syn + Cym19stop 21°C

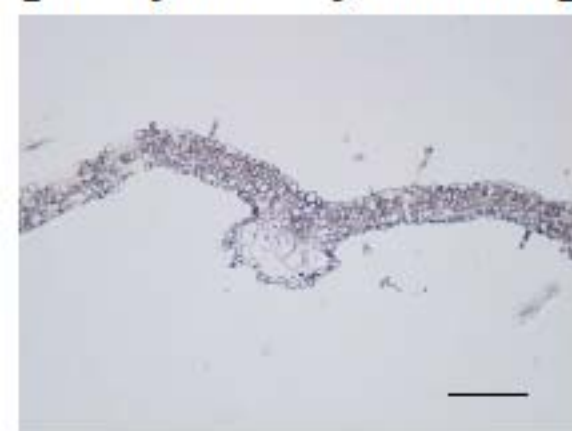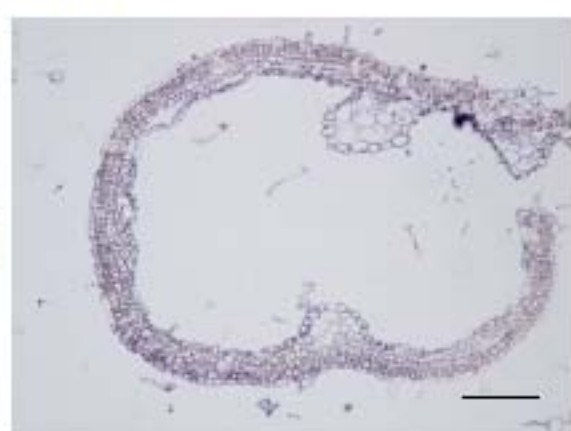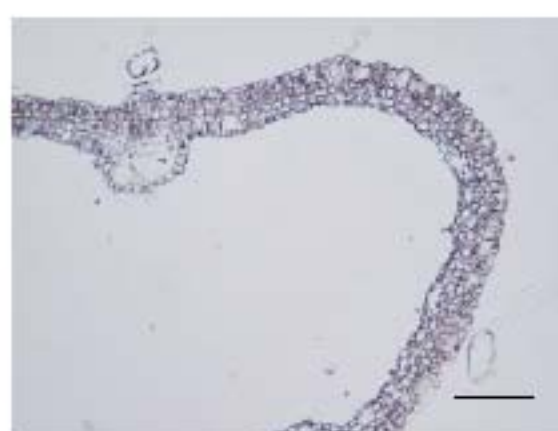

wt Nb + CymRSV 21°C

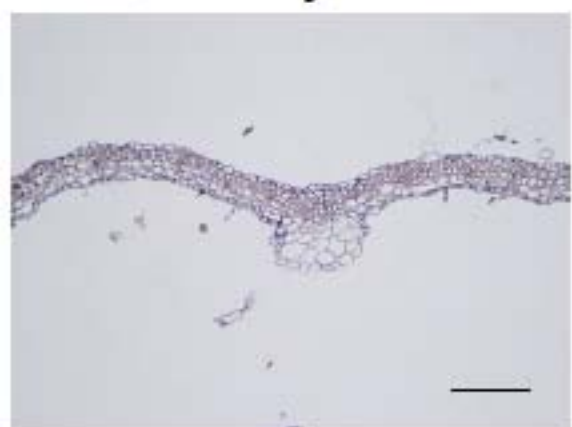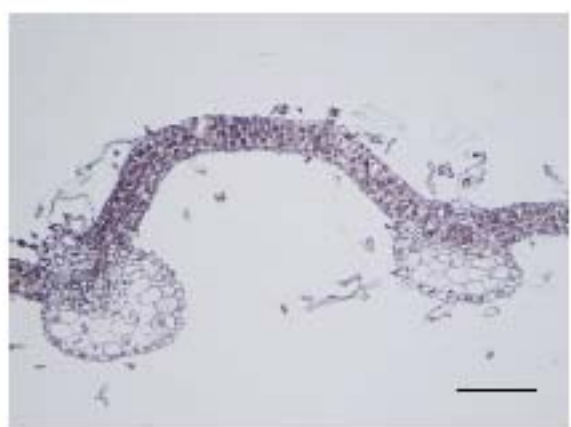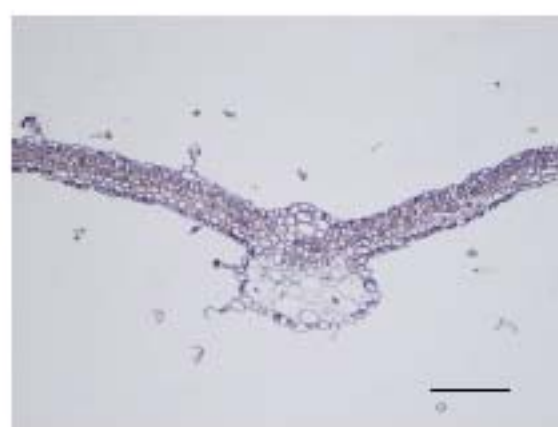

Bar= 200  $\mu$ m

Supplement: S2 Fig — (A) Characteristic phenotype of p19syn (1–57 line)(blue arrow) and virus-induced sytemic symptoms on wt or p19syn plants (red arrow) are shown. Pictures were taken at 10 dpi. (B) In situ hybridization of Cym19stop-infected wt N. benthamiana and p19syn plants or CymRSV-infected wt N. benthamiana leaf cross sections showing distribution of virus within the leaf tissue. Mock-infected wt N. benthamiana cross section shown as negative control (on the right). (PDF) [file ppat.1005935.s002.pdf]

S3 Figure

A

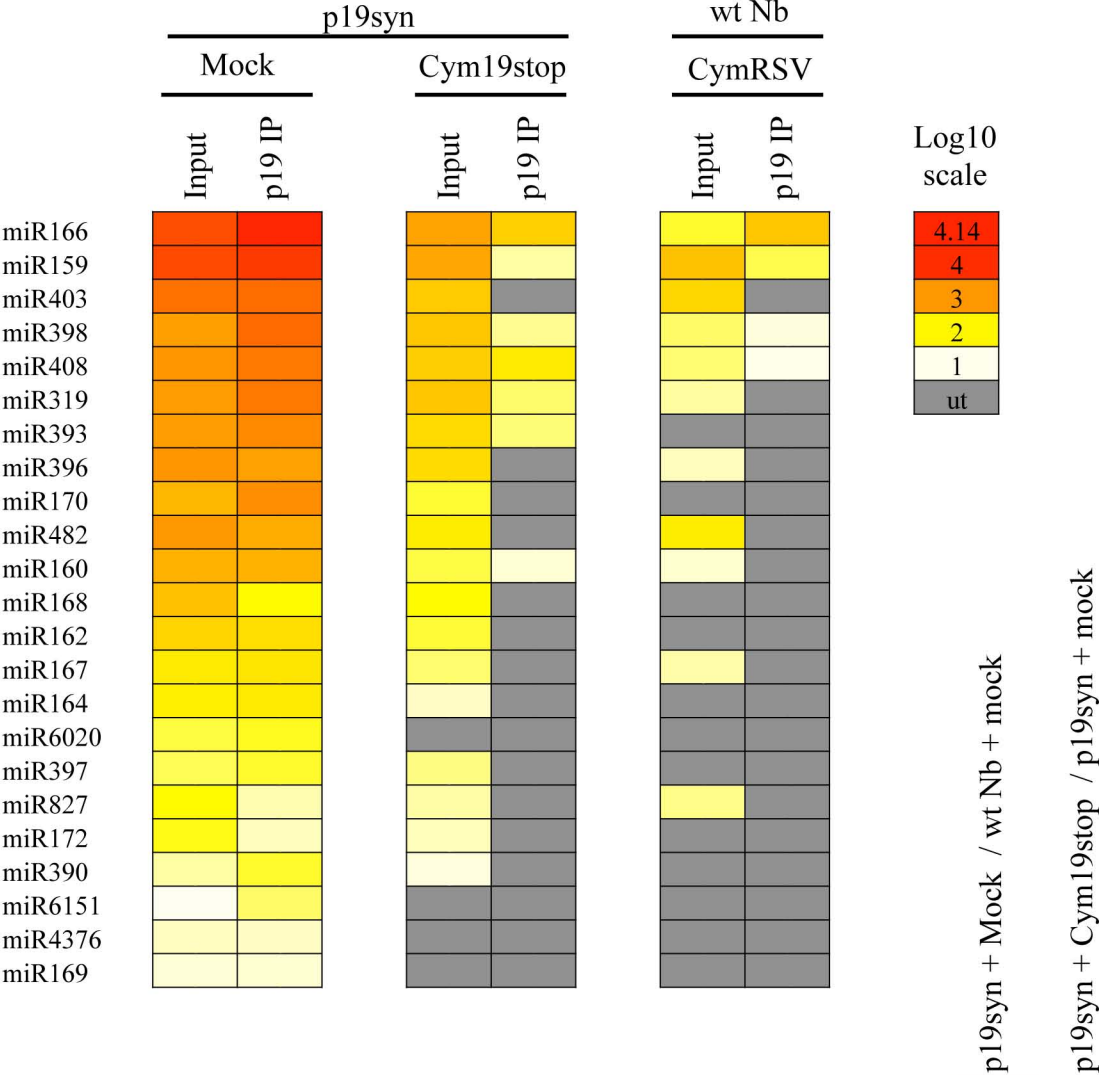

B

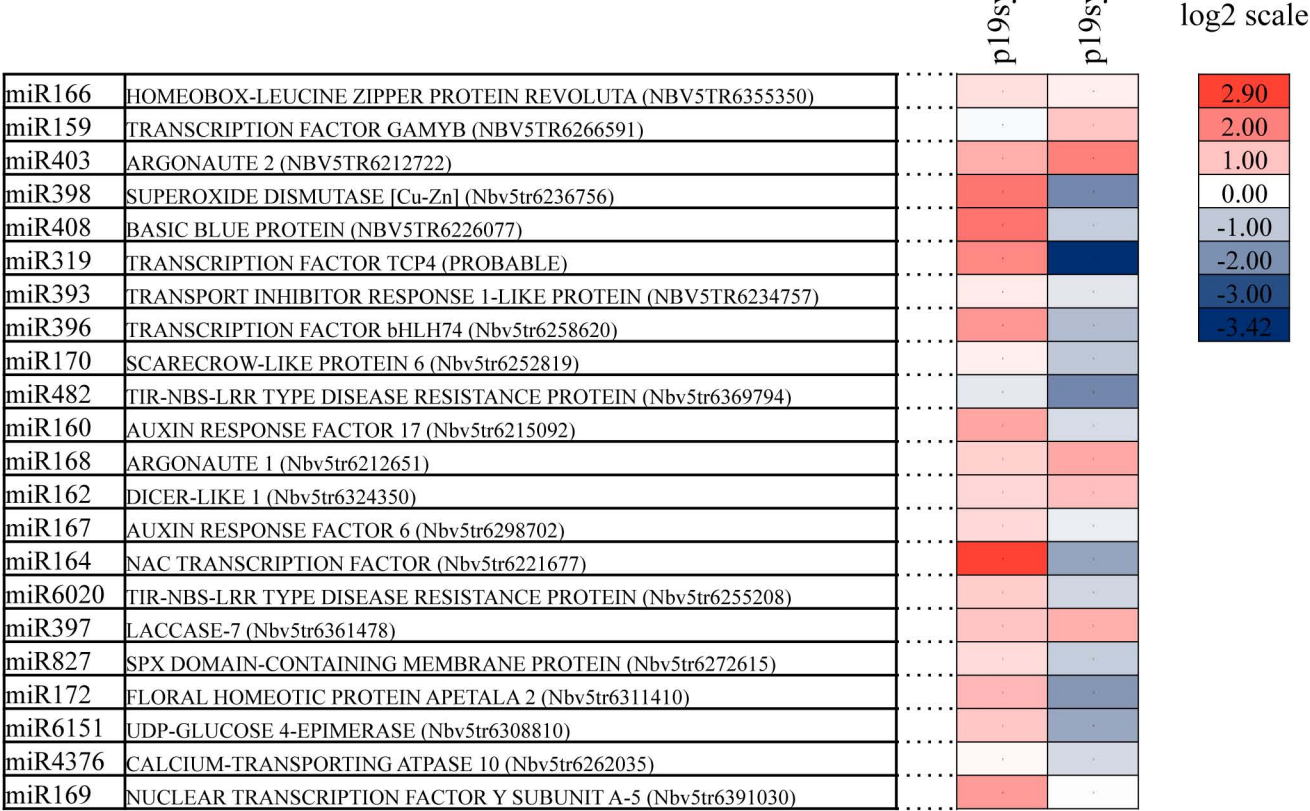

S3C Figure

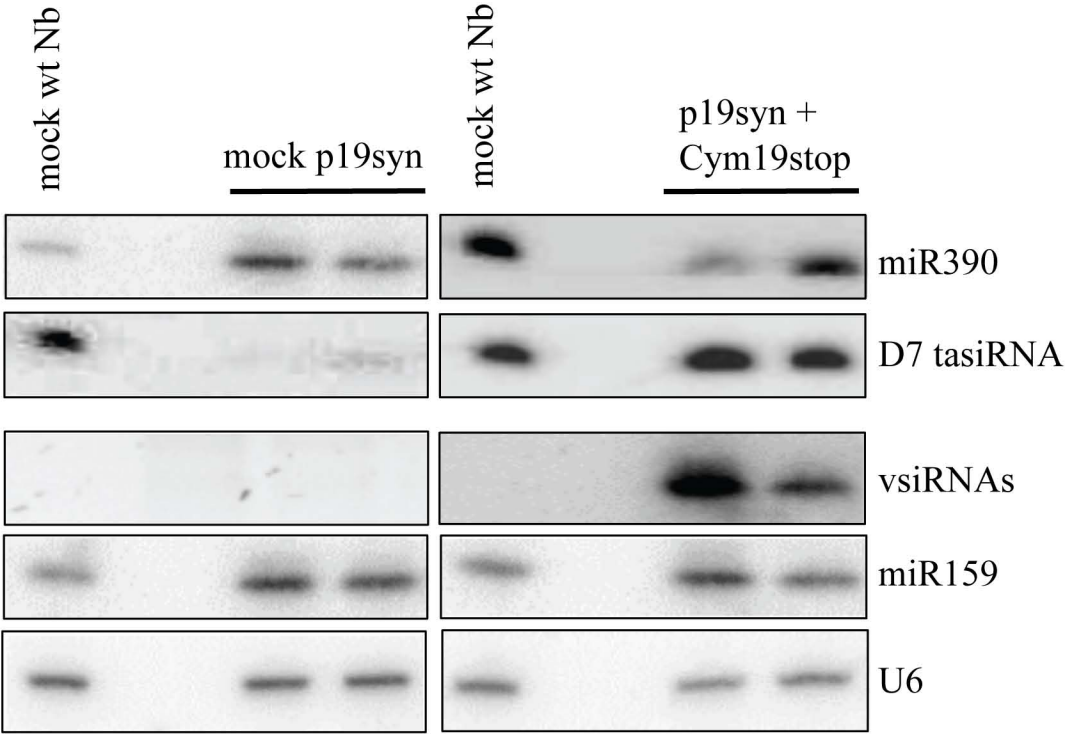

Supplement: S3 Fig — (A) Abundance of p19-bound miRNAs (listed on the left) in mock- or Cym19stop-infected p19syn plants and in CymRSV-infected wild type plants. The normalized total reads are shown on a log10 scale, values under threshold (ut). Heatmap legend is shown on the right. Read counts were normalized to 106 N. benthamiana genome matching read counts. (B) Ratio of miRNA-targeted mRNAs in mock-infected p19syn plants (relative to mock-infected wt N. benthamiana) and Cym19stop-infected p19syn plants (relative to mock-infected p19syn plants). Heatmap legend is shown on the right on a log2 scale. (C) Accumulation of miR390 and TAS3-derived D7 tasiRNAs in mock-infected or Cym19stop-infected p19syn plants compared to mock-infected wt N. benthamiana plants. vsiRNAs, miR159, and nucleolar small RNA U6 are shown as controls. (PDF) [file ppat.1005935.s003.pdf]

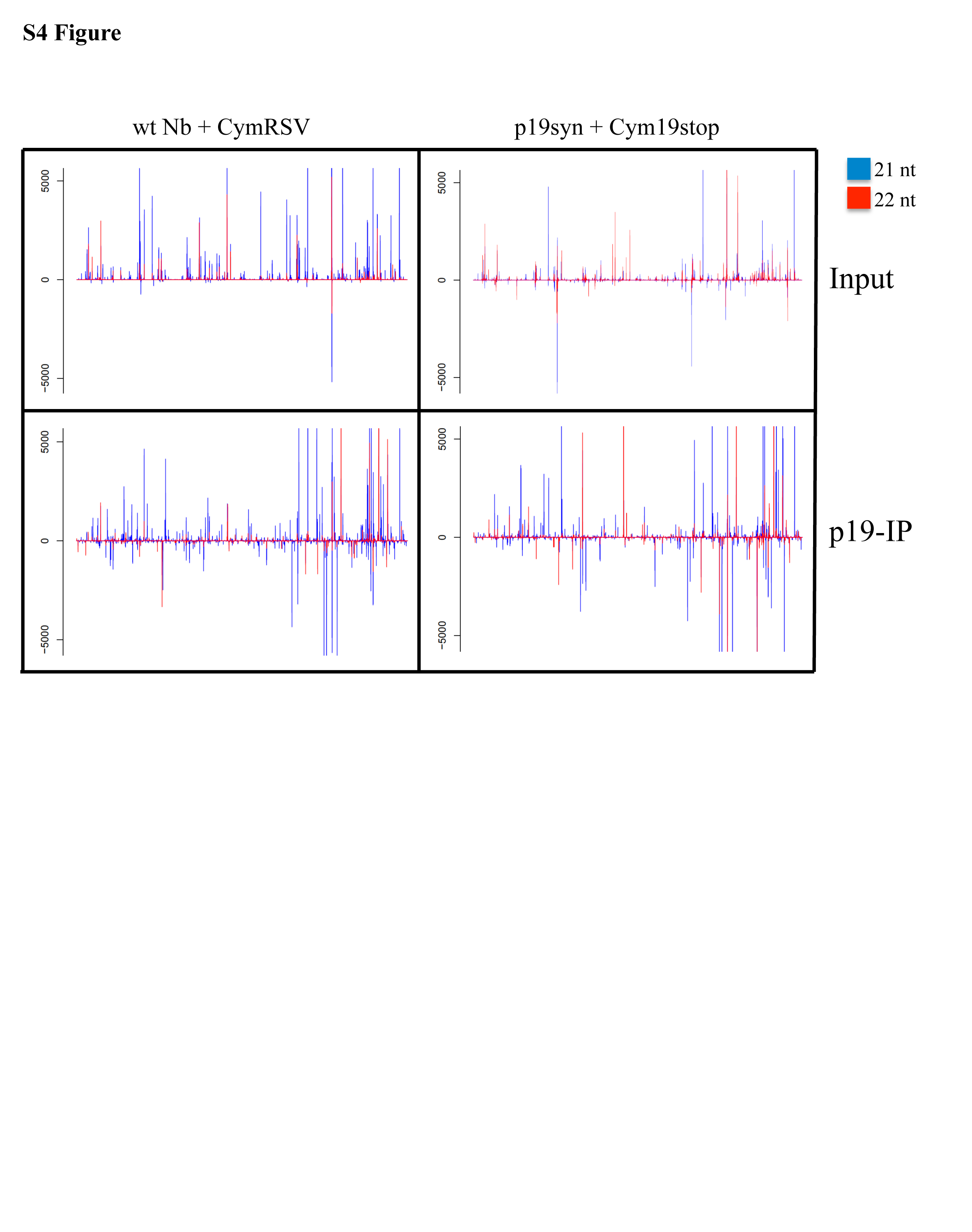

Supplement: S4 Fig — Location of vsiRNAs on the viral genome is presented on the x-axis, read counts are shown on the y-axis. Y-axis positive and negative values represent read counts derived from the positive or negative viral strand, respectively. 21nt and 22nt vsiRNAs are indicated in blue and red, respectively. Read counts were normalized to 106 “trimmed” read counts. (TIF) [file ppat.1005935.s004.tif]

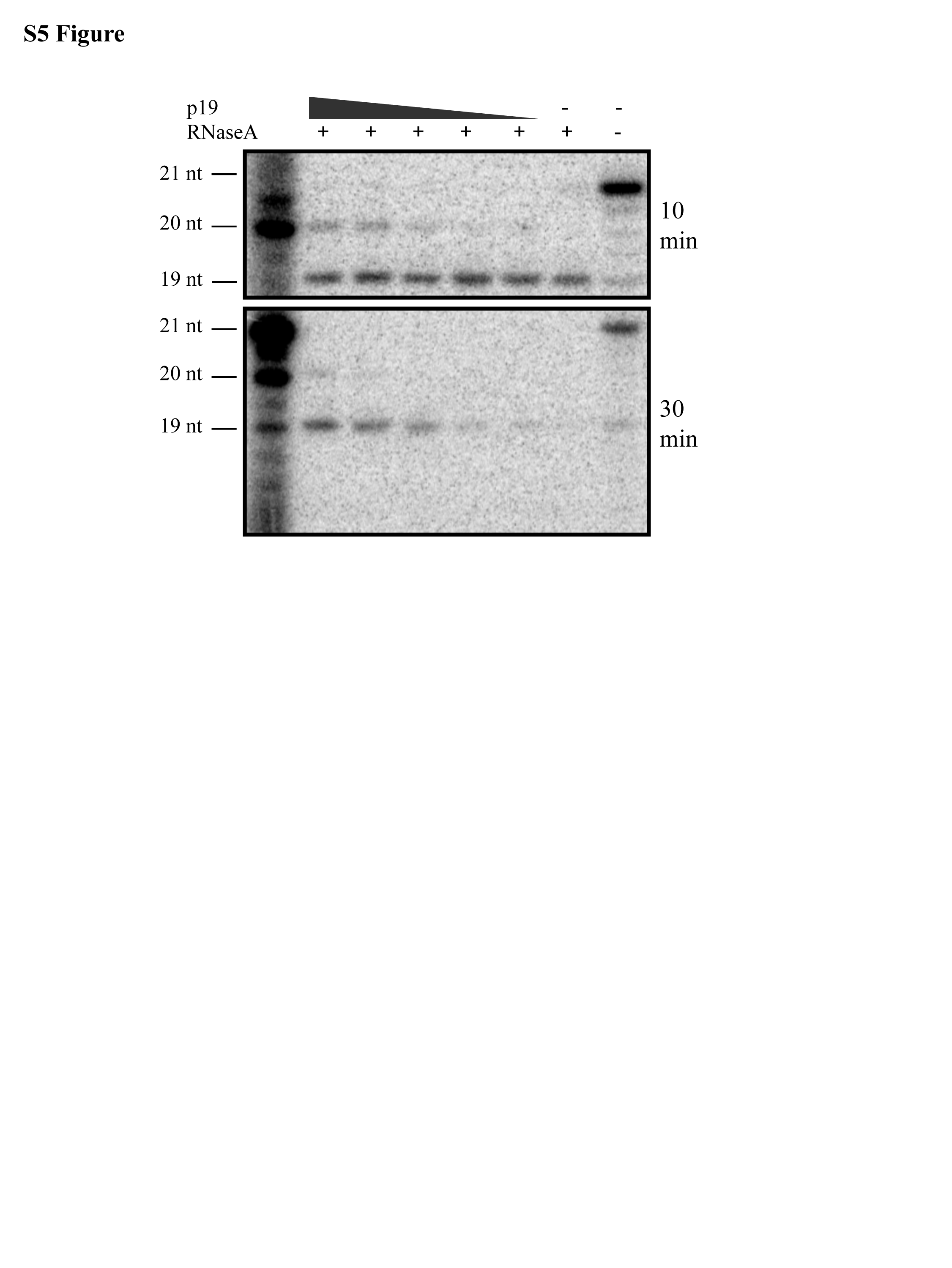

Supplement: S5 Fig — 5’ labeled siRNAs were incubated for 10 minutes with different amout of p19 and then exposed to RNase A-digestion for 10 and 30 minutes, upper and lower panel, respectively. The protected p19-bound RNA duplexes were analyzed on 16% denaturing acrylamide gel. Size marker is shown on the left. (TIF) [file ppat.1005935.s005.tif]

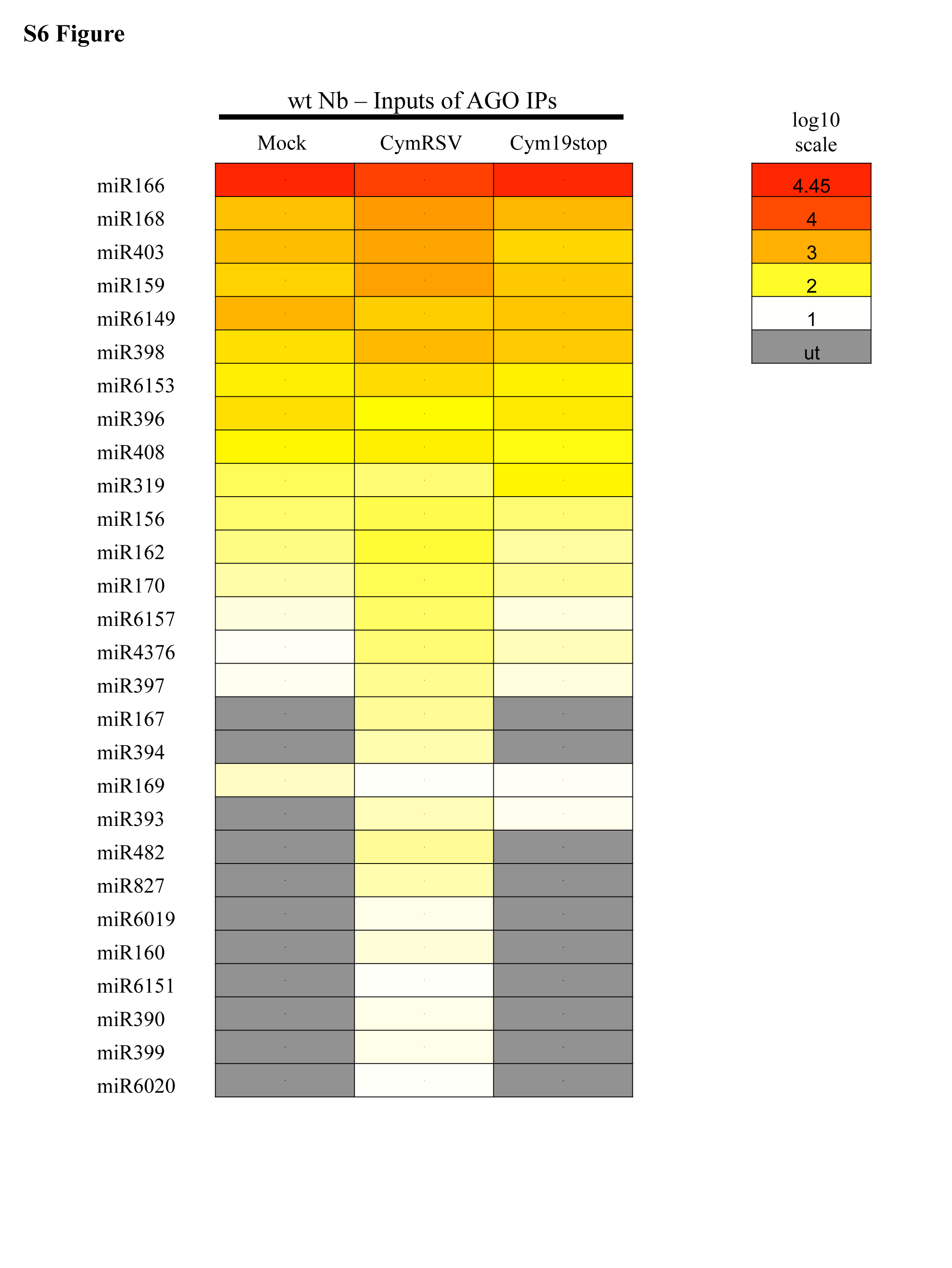

Supplement: S6 Fig — miRNAs analyzed are listed on the left. The normalized read counts values are shown on a log10 scale, values under threshold (ut). Read counts were normalized to 106 N. benthamiana genome matching read counts. (TIF) [file ppat.1005935.s006.tif]

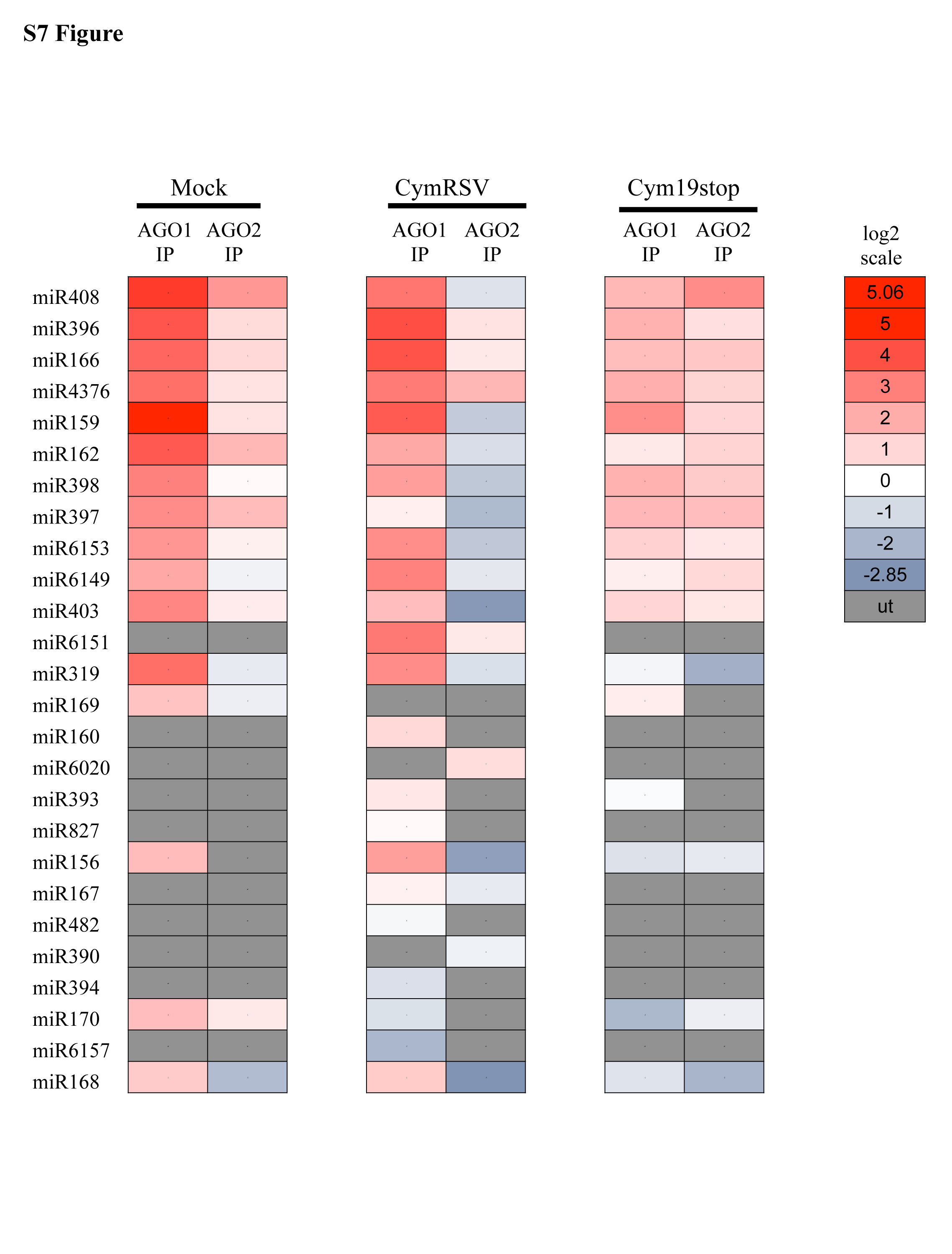

Supplement: S7 Fig — Abundance of miRNAs (listed on the left) in IP fraction were calculated relative to their inputs. The normalized values are shown on a log2 scale. Heatmap is shown on the right. (TIF) [file ppat.1005935.s007.tif]

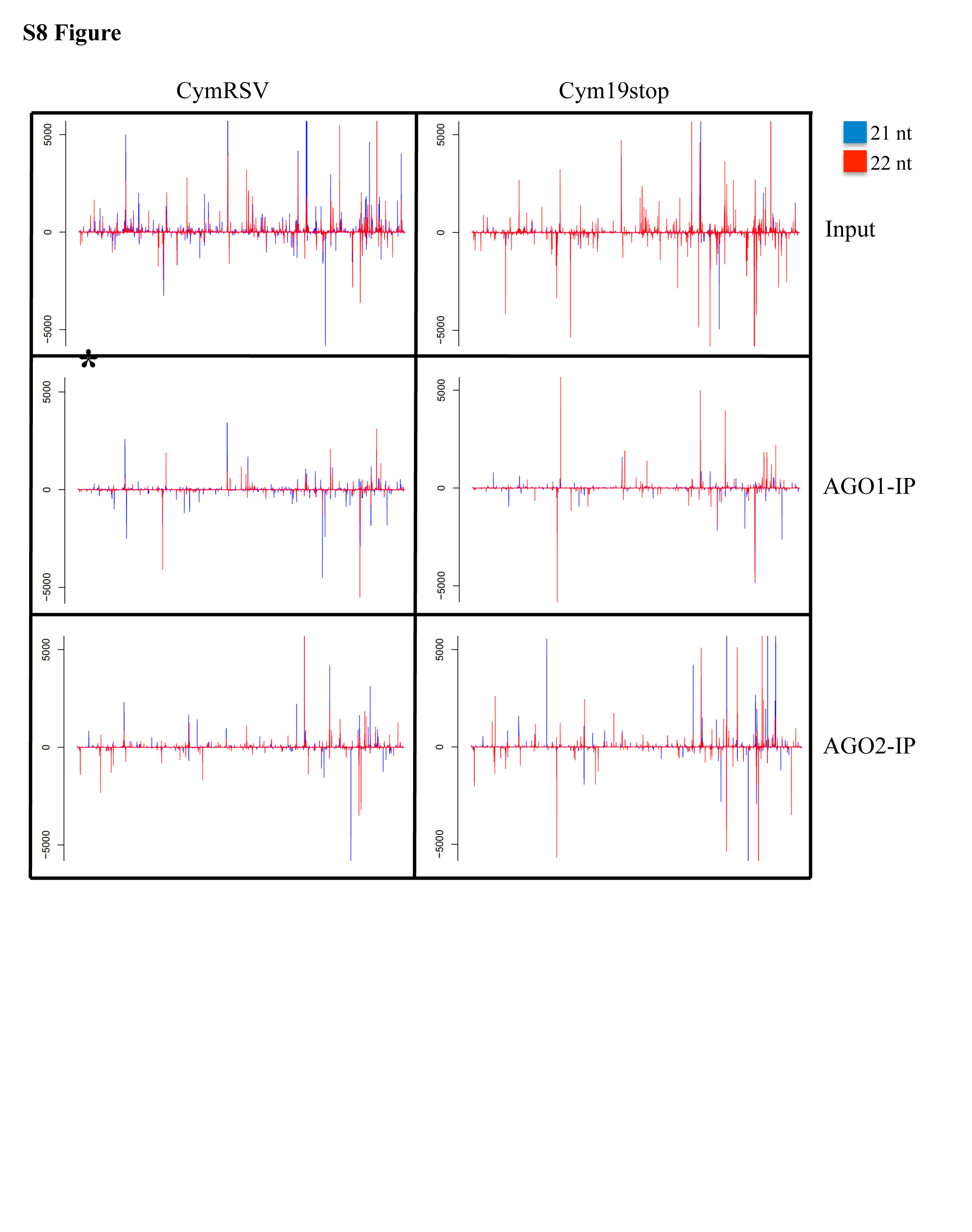

Supplement: S8 Fig — Location of vsiRNAs on the viral genome is presented on the x-axis, read counts are shown on the y-axis. Y-axis positive and negative values represent read counts derived from the positive or negative viral strand, respectively. 21nt and 22nt vsiRNAs are indicated in blue and red, as shown. vsiRNAs in AGO1-IP derived from CymRSV-infected plants is likely due to the nonspecific background as discussed in the text (black asterisk). Read counts were normalized to 106 total read counts. (TIF) [file ppat.1005935.s008.tif]

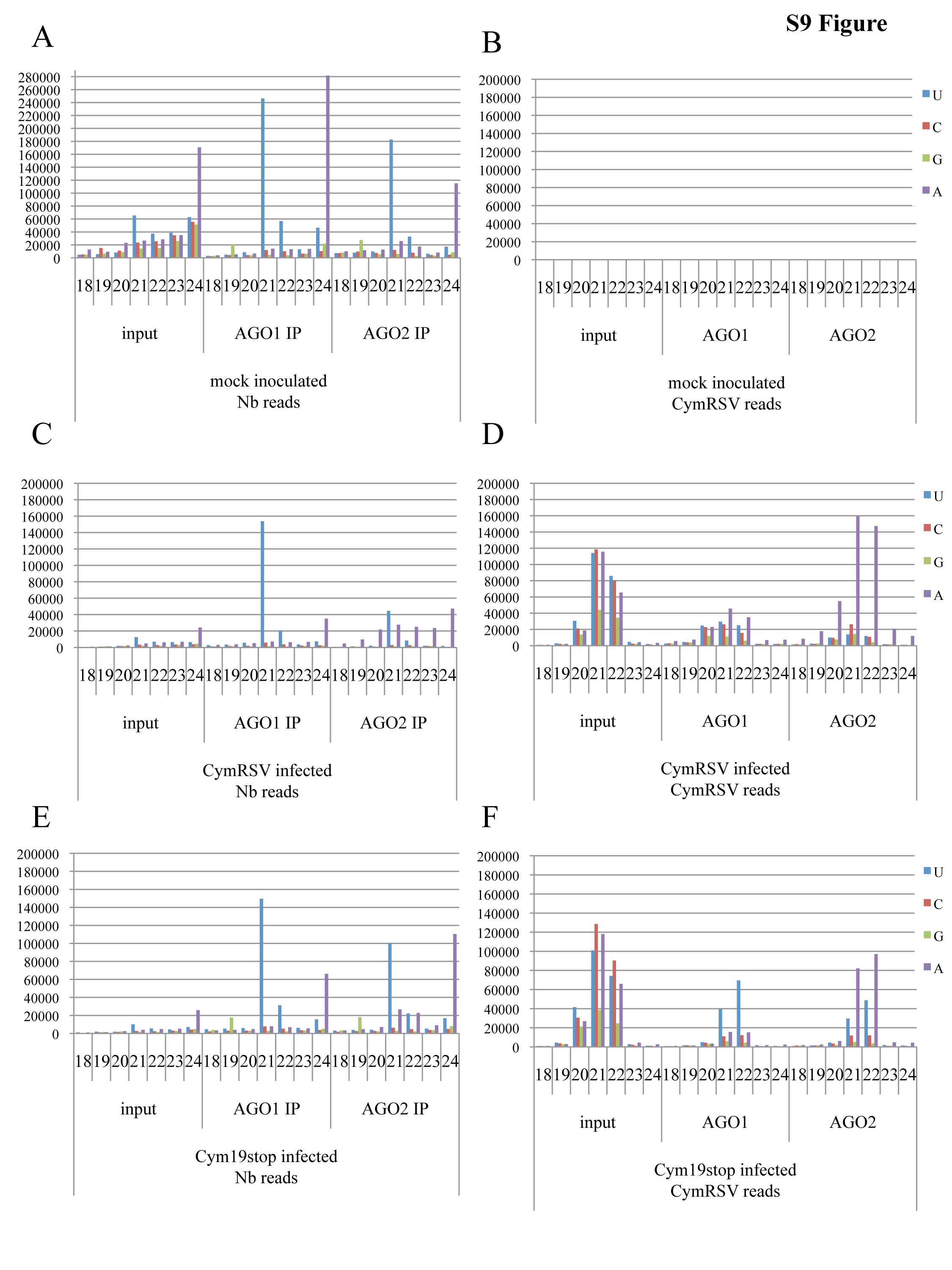

Supplement: S9 Fig — N. benthamiana specific reads from mock-inoculated (A), CymRSV- (C) and Cym19stop-infected wild-type plants (E). The vsiRNA reads of the same samples are presented in (B), (D) and (F), respectively. 5’ nucleotides of vsiRNAs are indicated by color code on the right. Size classes of sRNAs are indicated by numbers. Read counts were normalized to 106 total reads. Note the different scale in panel A. (TIF) [file ppat.1005935.s009.tif]

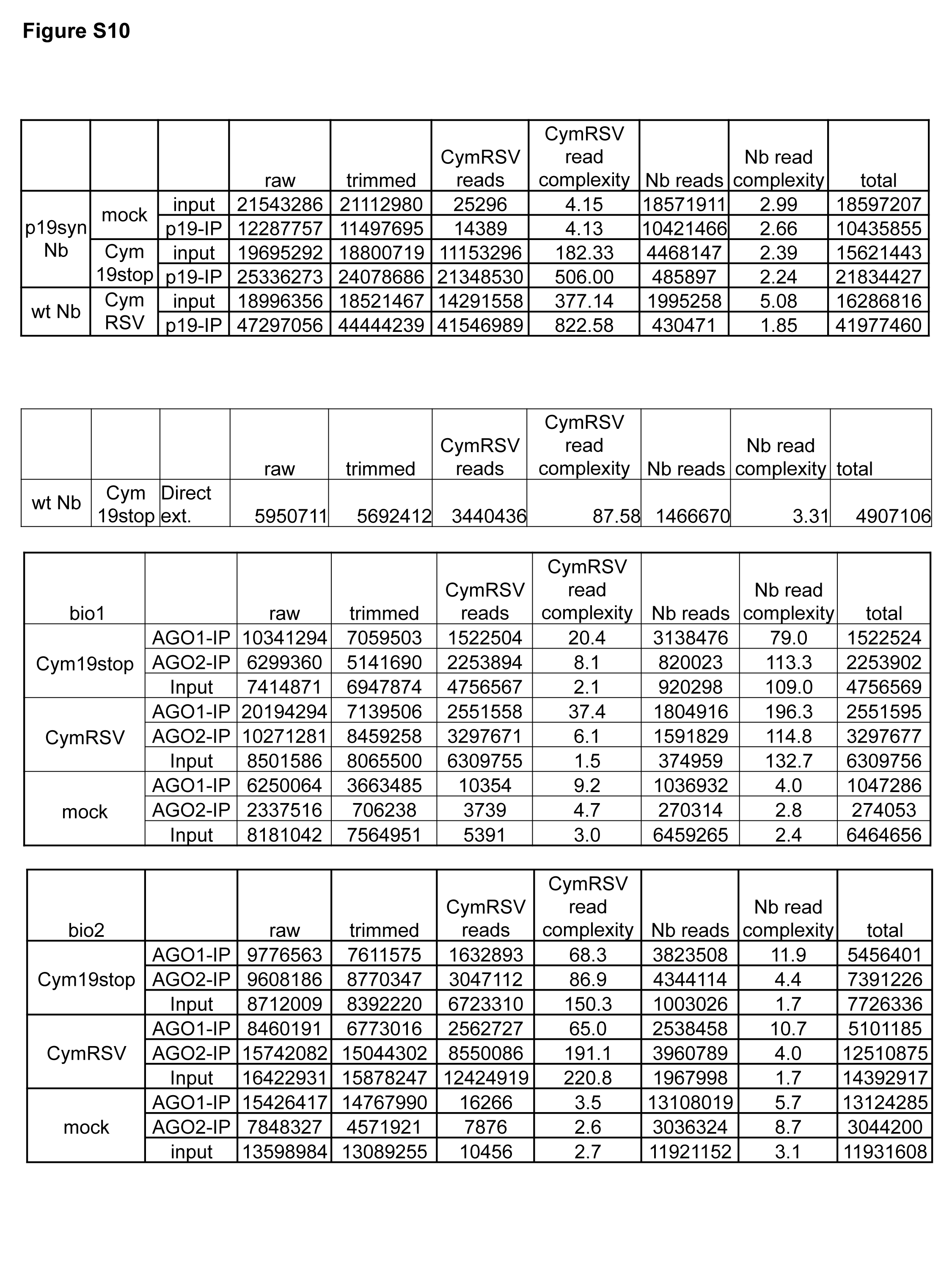

Supplement: S10 Fig — For detailed data processing information, please see Materials and Methods. (TIF) [file ppat.1005935.s010.tif]

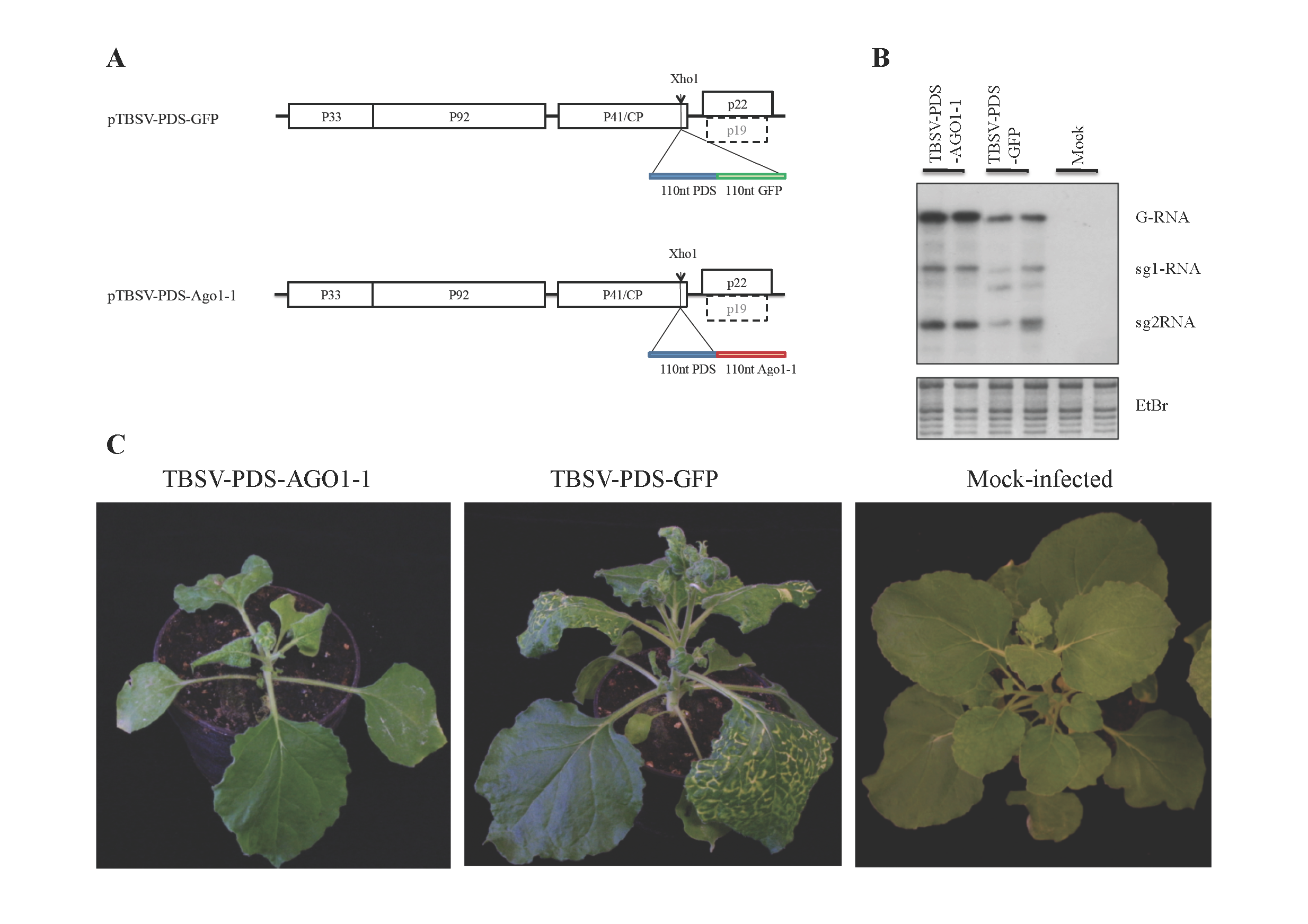

Supplement: S11 Fig — (A) Genomic organization of pTBSV-PDS-GFP and pTBSV-PDS-NbAGO1-1 VIGS constructs. (B) TBSV virus accumulation in NbAGO1-silenced, GFP-silenced and mock-infected (negative control) plants. (C) GFP-silenced but not NbAGO1-silenced plants show fast recovery phenotype. (TIFF) [file ppat.1005935.s011.tiff]
